# Refining circumstances of mortality categories (COMCAT): a verbal autopsy model connecting circumstances of deaths with outcomes for public health decision-making

**DOI:** 10.1080/16549716.2021.2000091

**Published:** 2022-04-04

**Authors:** Lucia D’Ambruoso, Jessica Price, Eilidh Cowan, Gerhard Goosen, Edward Fottrell, Kobus Herbst, Maria van der Merwe, Jerry Sigudla, Justine Davies, Kathleen Kahn

**Affiliations:** a Aberdeen Centre for Health Data Science (ACHDS), Institute of Applied Health Sciences, School of Medicine, Medical Sciences and Nutrition, University of Aberdeen, Scotland; b Department of Epidemiology and Global Health, Umeå University, Umeå, Sweden; c MRC/Wits Rural Public Health and Health Transitions Research Unit (Agincourt), School of Public Health, Faculty of Health Sciences, University of the Witwatersrand, Johannesburg, South Africa; d Public Healtlh, National Health Service (NHS), Scotland; e School of Geosciences, College of Science and Engineering, University of Edinburgh, Scotland; f Mpumalanga Department of Health, South Africa; g Institute for Global Health, University College London, UK; h Africa Health Research Institute, Durban, South Africa; i DSI-MRC South African Population Research Infrastructure Network (SAPRIN), South Africa; j Independent Consultant, South Africa; k Institute for Applied Health Research, University of Birmingham, UK; l International Network for the Demographic Evaluation of Populations and Their Health (Indepth), Accra, Ghana

**Keywords:** Causes of death, circumstances of mortality, verbal autopsy, South Africa

## Abstract

**Background:**

Recognising that the causes of over half the world’s deaths pass unrecorded, the World Health Organization (WHO) leads development of Verbal Autopsy (VA): a method to understand causes of death in otherwise unregistered populations. Recently, VA has been developed for use outside research environments, supporting countries and communities to recognise and act on their own health priorities. We developed the Circumstances of Mortality Categories (COMCATs) system within VA to provide complementary circumstantial categorisations of deaths.

**Objectives:**

Refine the COMCAT system to (a) support large-scale population assessment and (b) inform public health decision-making.

**Methods:**

We analysed VA data for 7,980 deaths from two South African Health and Socio-Demographic Surveillance Systems (HDSS) from 2012 to 2019: the Agincourt HDSS in Mpumalanga and the Africa Health Research Institute HDSS in KwaZulu-Natal. We assessed the COMCAT system’s reliability (consistency over time and similar conditions), validity (the extent to which COMCATs capture a sufficient range of key circumstances and events at and around time of death) and relevance (for public health decision-making).

**Results:**

Plausible results were reliably produced, with ‘emergencies’, ‘recognition, ‘accessing care’ and ‘perceived quality’ characterising the majority of avoidable deaths. We identified gaps and developed an additional COMCAT ‘referral’, which accounted for a significant proportion of deaths in sub-group analysis. To support decision-making, data that establish an impetus for action, that can be operationalised into interventions and that capture deaths outside facilities are important.

**Conclusions:**

COMCAT is a pragmatic, scalable approach enhancing functionality of VA providing basic information, not available from other sources, on care seeking and utilisation at and around time of death. Continued development with stakeholders in health systems, civil registration, community and research environments will further strengthen the tool to capture social and health systems drivers of avoidable deaths and promote use in practice settings.

## Background

‘Nothing exists until it is measured’. – Niels Bohr, 1930

Half the world’s deaths, approximately 30 million deaths per annum, pass without formal registration of medical cause [1]. Unregistered deaths are not randomly distributed around the world. In many low- and middle-income countries (LMICs), there are few physicians and certification of death is often lacking especially in disadvantaged and remote areas. Civil registration and vital statistics (CRVS) systems can also be incomplete or totally absent. Without continuous, valid and reliable mortality data, health care services can become, albeit inadvertently, configured to *maintain* social exclusion by failing to account for the situations and needs of those missed by state health and registration systems.

The *unequal world of health data* can be considered a social problem (such as crime and racism) [2]. Lack of data on the most fundamental aspects on people’s lives reflects and exacerbates structural inequalities, eroding rights beyond health, into security and citizenship. The situation raises important questions about the relationship between material and data poverty [3–6].

Verbal autopsy (VA) is currently the only realistic alternative in settings where medical certification of deaths is rare, incomplete or absent. VA is a pragmatic survey-based method in which trained fieldworkers gather information from final caregivers on signs and symptoms of the deceased prior to death. From these data, probable medical cause(s) of death are separately interpreted. In sufficient numbers, the technique can reliably quantify levels and causes of death in otherwise unregistered populations. VA has been used in over 45 LMICs for more than two decades, mainly in research and demographic surveillance settings, to understand disease burdens and health transitions [8],

The World Health Organization (WHO) leads development of international standards for VA. In recent years, there have been major advances: from physician-coding of VA interview data to automated computer coding using mobile technologies. Acceptable, reproducible and scale-able approaches reflect significant, collective global action to provide information on otherwise unknown mortality [8–14]. Informing and driving these shifts, the WHO has directed development of VA to support and enable countries and communities to understand and act on their own health priorities. In 2012, the international VA standard for the first time focussed on large scale automated application widening routine, large-scale implementation outside research environments and in CRVS systems [15].

Simultaneously, a growing body of work recognises the value of routinely recording information on circumstantial factors contributing to deaths. Indicators on care processes such as case detection, treatment, service availability, access and met need have been used to understand service responses in connection with outcomes [16,17]. Other methods, such as confidential enquiries and social autopsy, provide detailed information on modifiable factors, e.g. service utilisation and health systems responsiveness, in connection with outcomes [18,19]. Frameworks locating medical outcomes within the social, cultural and health systems landscapes in which care is situated and sought can inform health planning cognisant of the deeper drivers of avoidable mortality [20]. While the social determinants of heath are accepted as the fundamental causes of avoidable mortality and health inequalities, however, systematic and scalable circumstantial categorisations of deaths have not been developed [20].

This paper reports on developing VA to account for social and health systems circumstances of deaths, along with medical causes, in a way that supports data for action. For example, a woman whose cause of death is assigned as obstetric haemorrhage might have died at home, while another woman with the same cause of death might have been inadequately managed despite reaching a facility. Measuring these scenarios at population level will provide important information for health services and reducing avoidable mortality. We previously devised an approach within VA tools called Circumstances of Mortality Categories (COMCAT) [21]. Not intended to replace social autopsy, the COMCAT system is designed for large-scale population assessment of burden of disease inclusive of the needs and behaviours of individuals, and the responsiveness of the health system towards these [22].

In developing the COMCAT system, we formulated and field-tested input questions on care seeking and utilisation at and around the time of death [23], which were taken up in the 2012 WHO VA standard [15]. From this, we developed VA data interpretation tools to assign numeric likelihoods to circumstantial categories for each death on: ‘emergencies’, ‘recognition of severity’, ‘use of traditional medicine’, ‘mobilising resources to seek care’ and ‘problems with admission, treatment and care’ (Table 1). In 2019, we published a proof of principle analysing VA data from 4,116 deaths in the Agincourt Health and Socio-Demographic Surveillance System (HDSS) in South Africa 2012–16, which established the COMCAT system as consistent, reproducible and plausible [21].

**Table 1.** Questions/substantive responses on circumstances of mortality (critical limiting circumstances and events at and around the time of death related to the needs and behaviours of individuals and the responsiveness of the health system towards these) from WHO-2012 and WHO-2016 WHO VA standards and circumstances of mortality categories (COMCATs)

**(a) WHO-2016 question/item**	**Explanation of substantive responses**
Id10450 In the final days before death, did she/he travel to a hospital or health facility?	A ‘no’ response indicates no contact with hospital-level services in the days before death (defined as a 24/7 service, but noting in some settings 24/7 facilities may not be called ‘hospitals’).
Id10451 Did she/he use motorised transport to get to the hospital or health facility?	A ‘yes’ response indicates that the person who died travelled to a hospital or health facility by means of motorised transport (car, truck, tractor, motorcycle, scooter or ambulance) during the final illness.
Id10452 Were there any problems during admission to the hospital or health facility?	A ‘yes’ response indicates that the person who died travelled to a hospital or health facility, but then had problems on arrival (delays, paperwork, queues, no staff).
Id10453 Were there any problems with the way she/he was treated (medical treatment, procedures, inter personal attitudes, respect, dignity) in the hospital or health facility?	A ‘yes’ response indicates that the person who died travelled to a hospital or health facility, but then had problems with how they were treated (medical treatment, procedures, inter-personal attitudes, respect, dignity).
Id10454 Were there any problems getting medications, or diagnostic tests in the hospital or health facility?	A ‘yes’ response indicates that the person who died travelled to a hospital or health facility, but then had problems obtaining essential items (drugs, medications or other prescriptions, blood products, and/or diagnostic tests such as lab tests and X-rays, either within the facility or needing to be bought elsewhere).
Id10455 Does it take more than 2 hours to get to the nearest hospital or health facility from the deceased’s household?	A ‘yes’ response indicates that the person who died lived in a household from where it would reasonably take more than 2 hours to reach the nearest 24-hour health facility by the means of transport available to the household members.
Id10456 In the final days before death, were there any doubts about whether medical care was needed?	A ‘yes’ response indicates that there were doubts among those assisting in the final illness (family members, etc.) about whether the final illness was sufficiently serious to need treatment at a health facility.
Id10457 In the final days before death, was traditional medicine used?	A ‘yes’ response indicates that a major part of treatment for the final illness was provided by any kind of traditional or alternative practitioner (herbal remedies, massages, drinks, foods, amulets, spells or blessings provided by traditional healers, witch doctors or shamans).
Id10458 In the final days before death, did anyone use a telephone or cell phone to call for help?	A ‘no’ response indicates that no telephone of any kind (working landline, or cell phone charged and with credit) was used by those assisting in the final 24 hours of the illness, for example to call for help or arrange transportation.
Id10459 Over the course of illness, did the total costs of care and treatment prohibit other household payments?	A ‘yes’ response indicates that the total costs incurred in the final illness were sufficiently great to mean that other kinds of household expenses (food, fuel, travel, education etc.) could not be met, or caused debt or sale of household assets.
**(b) COMCAT**	**Description of circumstantial category**
Traditions	Traditional practices or beliefs influenced health seeking behaviour and the pathway to death.
Emergencies	Sudden, urgent or unexpected conditions leading to death.
Recognition	Lack of recognition or awareness of serious disease (e.g. symptoms or severity) negatively influenced health seeking behaviour.
Resources	Inability to mobilise and use resources (e.g. material, transport, financial) hindered access to health care.
Health systems	Problems in getting health care despite accessing health facilities (e.g. related to admissions, treatments and medications).
Inevitability	Death occurred in circumstances that could not reasonably have been averted (e.g. very elderly or recognised terminal conditions).
Multiple	A combination of the above categories affected the pathway to death; no single factor predominated.

Circumstances of mortality have since been used to understand barriers to access in injuries, trauma and in time-critical conditions [24,25], and COMCAT has been taken up in a national cause of death study in South Africa [26]. Acknowledging further development as a priority, in this study, we evaluated and refined the COMCAT system with stakeholders from Mpumalanga Department of Health, South Africa, as part of a wider research programme (www.vapar.org). The objectives were to (a) refine COMCAT as a pragmatic and scalable approach for large-scale population assessment of critical limiting circumstances of deaths without adding to the burden of VA; and (b) appraise its capability to inform public health decision-making.

## Methods

### Context and population

South Africa is an upper-middle-income country with a population of 59.6 million, a third of which is rural. Life expectancy was 66 years in 2020 [27]. South Africa is one of the most unequal countries in the world. HIV prevalence in black populations is 40–50 times that of white, and HIV risks are eight times higher in young women compared to men of the same age [28,29]. The country has a complex burden of communicable and non-communicable mortality, with injury and violence mortality double the global average [3,28,30]. While health policy is progressive and inclusive, entrenched systems challenges (including with investment, human resources and governance) significantly limit progress [31–33].

We analysed VA data from two rural HDSS nodes within the South African Population Research Infrastructure Network (SAPRIN), where information on circumstances of deaths had been recorded since 2012. Firstly, in Mpumalanga province, the Agincourt HDSS has 116,549 people under surveillance, 33% of which are under 15 years old. In the population, there is high unemployment, limited sanitation and underdevelopment [34,35]. Secondly, in KwaZulu-Natal province, the Africa Health Research Institute (AHRI) has 139,250 people under surveillance of which 32% are under 15 years old. The surveillance population is among the lowest-ranked in the country in terms of socioeconomic status with high unemployment rates and limited access to piped water [35–39].

### Data analysis

As described above, we previously developed VA input indicators on circumstances of mortality conceived of as: critical limiting circumstances and events at and around the time of death related to the needs and behaviours of individuals and the responsiveness of the health system towards these. The indicators were adopted in WHO-2012 VA standard (Table 1a) [15]. Since 2012 and 2017 in the Agincourt and AHRI HDSSs, respectively, VA questionnaires have been based on this standard and on subsequent versions.

VA data inclusive of circumstances indicators were obtained from the HDSSs and a harmonised naming Table 2 and coding system was developed [40]. All data were processed using InterVA-5 (version 5.1). InterVA-5 is a widely applied and evaluated machine algorithm to interpret VA data. It is freely available, open source, aligned to WHO standards, other VA models, and HDSS and CRVS infrastructure globally [41]. We used InterVA-5 because the COMCAT system was developed within it, and no other automated model currently has this functionality [21].

**Table 2.** Circumstances of mortality categories (COMCATs), revised

COMCAT	Description of circumstantial category
Traditions	Traditional practices or beliefs influenced health seeking behaviour and the pathway to death.
Emergencies	Sudden, urgent or unexpected conditions leading to death.
Recognition	Lack of recognition or awareness of serious disease (e.g. symptoms or severity) negatively influenced health seeking behaviour.
Accessing care	Inability to mobilise and use resources (e.g. material, transport, financial) hindered access to health care.
Perceived quality	Problems in getting health care despite accessing health facilities (e.g. related to admissions, treatments and medications).
Referral	Problems receiving a referral when required. Problems reaching referral facility after referral made.
Inevitability	Death occurred in circumstances that could not reasonably have been averted (e.g. very elderly or recognised terminal conditions).
Multiple	A combination of the above categories affected the pathway to death; no single factor predominated.

For each death, the InterVA model applies Bayes theorem and a set of prior probabilities relating input indicators to medical causes of death to calculate the probability of each cause. In prior work, InterVA-5 was developed to incorporate a sub-model that separately processes all indicators from the interview into likelihoods for COMCATs. If the likelihood of one of the six COMCATs exceeds 50%, that category is assigned to the case, otherwise, the ‘multiple’ category applies [42]. We revised two COMCAT labels to better reflect the circumstantial category being captured without changing the content of the categories: ‘resources’ (mobilising resources to seek care) was updated to ‘accessing care’ and ‘health systems’ (problems experienced/perceived with admission, treatment and care) was updated to ‘perceived quality’. We also updated the description of ‘emergencies’ (Table 1b).

We evaluated the COMCAT system according to three criteria. Firstly, we evaluated *reliability*, defined as overall consistency over time and broadly similar conditions. Secondly, we assessed *validity*, defined as the extent to which the COMCAT system captures a sufficiently complete range of critical limiting circumstances and events at and around the time of death. Thirdly, we appraised *relevance*, defined as the ability to generate information for public health decision-making. Refinements and recommendations were then formulated.

We assessed *reliability* by reproducing the 2019 proof-of-concept analysis, examining cause and age-specific mortality in relation to COMCATs over an extended time period and in another setting. We re-produced ‘heatmaps’ assigning precise proportions of deaths attributed to each COMCAT for the major cause of death group and for all deaths. We also calculated cause and COMCAT-specific burdens in absolute terms by age group and over time.

To assess *validity*, we mapped the current COMCATs onto the Pathways to Survival Framework [43]. This framework has been widely used to identify contributory factors in care provided in the home and sought/received outside the home for neonatal and under-5 mortality, and provides valuable insights into all-cause adult mortality [19,44–48]. Arranging the COMCATs relative to the framework enabled assessment of the entire range of factors captured and where gaps existed.

We then examined the circumstantial inputs to judge whether items could be removed so that additional inputs/outputs addressing gaps would not add to the burden of VA. This was a consensus process among a group of VA experts on the likelihood of questions deriving important information across settings. It was also informed by the data: how inputs were answered by respondents and used by the InterVA-5 model. Through group and individual discussions and written exchanges, we assessed the current input questions, identified candidates for removal and possible replacement questions from site-specific VA interviews in Agincourt and AHRI. On this basis, we modified the COMCAT sub-model within InterVA-5 to output an additional COMCAT addressing gaps identified. We applied the same conceptual and statistical methodology as used in the initial COMCAT concept: updating the probability matrix with medical professionals and researchers. We then ran and analysed a subset of data.

Finally, to appraise *relevance*, we held group and individual discussions and written exchanges with co-authors in Mpumalanga Department of Health. We interrogated and appraised the COMCAT system’s meanings, clarity and descriptions, gaps and refinements, developing different data presentations and visualisations for service planning and review [49]. This was supplemented with a simple content analysis of provincial and district health systems planning documents, examining how COMCATs corresponded with targets and indicators therein [50,51].

## Results

During the period 2012–19, collectively from Agincourt and AHRI, 7,980 deaths were observed and followed up with VA. Consistent with previous analysis [21], a high burden was attributed to non-communicable diseases (NCDs), infectious diseases and a significant burden of external mortality causes (injuries, accidents and assaults). 40.2% of all deaths were attributed to NCDs, 32.9% to infectious causes (of which 20.1% to HIV/AIDS and TB), 11.5% to external causes, and 2% to maternal and neonatal causes. 13.5% of deaths were of indeterminate cause. Sex-specific mortality (medical causes of death) was balanced (49.5% of deaths among females) in all causes other than injuries, accidents and assaults (76.9% among males) (Supplementary Material 1 includes site-specific results).

### Reliability

Most deaths were attributed to the COMCAT: ‘inevitability’ (20.2%), reflecting a large proportion of deaths over 70 years (28%). Otherwise, the COMCATs assigned with highest frequency were ‘emergencies’ (20.1%), ‘accessing care’ (18.6%), ‘perceived quality’ (17.9%) and ‘recognition’ (15.9%). COMCATs displayed some sex differences, in ‘emergencies’ (63.8% among males), ‘traditions’ (62.3% among females) and ‘recognition’ (57.2% among males).

**Figure 1.**
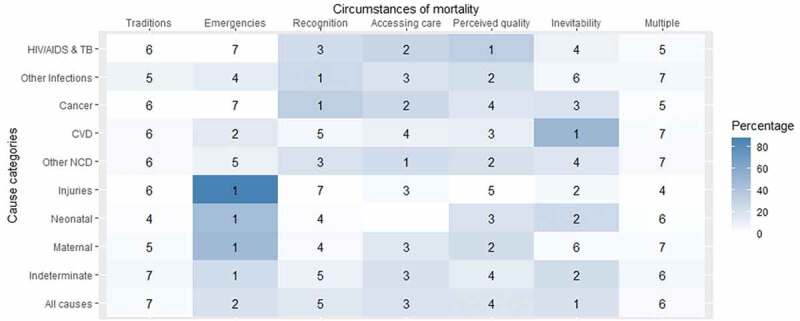
Assigned circumstances of mortality categories (COMCATs) ranked within each major cause of death category for 7980 deaths in the Agincourt and AHRI health and demographic surveillance systems (HDSSs) 2012–19 and 2017–19 respectively.

Consistent with the proof of concept analysis [21], COMCATs varied plausibly with medical cause of death. Injuries were strongly and plausibly associated with ‘emergencies’. HIV/AIDS and TB deaths were mainly attributed to ‘perceived quality’, which is conceivable considering problems with met need, enduring stigmatisation and high costs within and outside the health system. Other chronic conditions were associated with ‘accessing care’. This is also reasonable considering multiple presentations for care over the course of a chronic illness may limit the ability to mobilise resources to access care in the acute episode. Similar to the proof of concept analysis (21), small proportions of deaths were assigned to ‘multiple’ (3.8%) and ‘traditions’ (3.6%) (Figure 1, Supplementary Material 1).

**Figure 2.**
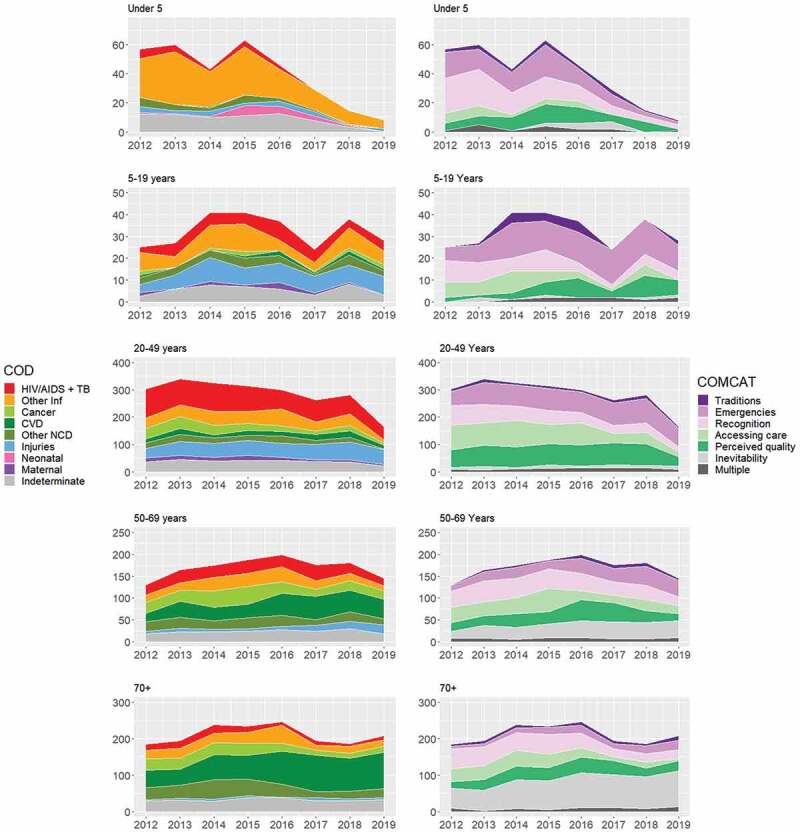
Number of deaths for cause-specific mortality fractions (CSMFs) and circumstances of mortality categories (COMCATs) stratified by year and age groups for 5924 deaths in the Agincourt HDSS 2012–19.

Figure 2 presents cause and COMCAT-specific burdens over time and by age group. Consistent with the proof of concept analysis [21], there was a substantial reduction in under-5 mortality driven by reductions in infectious diseases, together with reductions in all COMCATs. Among 20–49 year olds, there was a large decline in overall mortality, characterised by reductions in HIV and TB. Significant injury-related mortality was observed in this age group, however, with many deaths attributed to the ‘emergencies’ COMCAT. Among 5–19 year olds, while overall mortality was again relatively low, injury-related deaths increased, along with the ‘emergencies’ COMCAT. In the 50–69 years age group, deaths attributed to cardiovascular disease (CVD) increased, with concurrent increases in the ‘emergencies’ and ‘inevitability’ COMCATs. CVD also increased substantially in the 70-plus age group, with most deaths in this age group attributed to the ‘inevitability’ COMCAT.

### Validity

Mapping to the Pathways to Care framework illustrated that the COMCATs covered a range of modifiable factors: caregiver knowledge, accessing care outside the home, use of informal care, and health system factors contributing to perceptions of poor quality. However, we also identified that problems with referral and home care recommendations (health worker advice on giving medication at home, signs of deterioration and follow-up) were not captured (Supplementary Material 2). Considering the significance of these factors across LMICs [19,44,46–48,52], these aspects were considered gaps.

We then assessed the circumstantial inputs. Existing questions on *traditional medicine; prohibitive costs*; and *distance to facilities* were assessed to be worthy of retaining considering their importance across LMIC settings. For the questions on *treatment*; and *medication*, while acknowledging that respondents may neither witness clinical events nor be able to assess them [23], these aspects were considered sufficiently notable and important across settings. Inputs on *travelling to hospital; doubts about the need for care*; and *use of motorised transport* were also assessed to be worthy of retaining owing to high importance across settings. However, proposed rewordings were developed to identify problems with better accuracy. Finally, questions on *use of mobile phone* and *admission* were assessed to be outside the core focus and identified as candidates for removal. Overall, we identified five questions to retain, three to amend, and two to remove (Supplementary Material 3).

We then reviewed VA tools from the Agincourt and AHRI HDSSs, identifying two input items: *receiving a referral*; and *reaching a referral facility*, to address gaps identified. The InterVA-5 model was modified to output an additional COMCAT on ‘referral’ defined as ‘*Problems receiving a referral when required. Problems reaching referral facility after referral made’*. The ‘referral’ COMCAT was developed avoiding overlap with ‘accessing care’, which relates to problems mobilising resources to seek care (Table 2).

**Figure 3.**
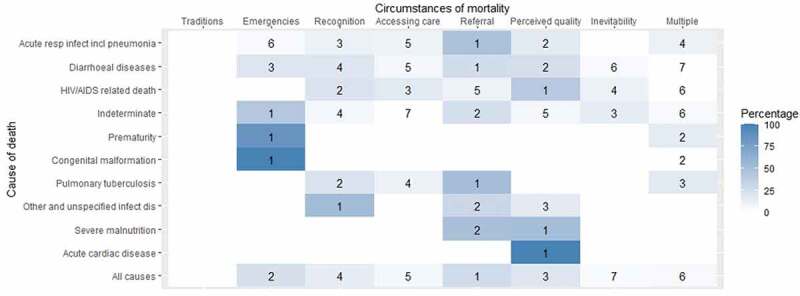
Assigned circumstances of mortality categories (COMCATs) ranked within top ten causes of those aged 12 and under for 105 deaths in the Agincourt and AHRI health and demographic surveillance systems (HDSSs) 2016–19 and 2017–19 respectively health and demographic surveillance systems (HDSSs) 2016–19.

In a sub-group analysis of 105 under-12 deaths from the Agincourt and AHRI HDSSs 2016–19 and 2017–19, the ‘referral’ COMCAT was attributed to 27.6% of deaths, representing the most common COMCAT for all-cause mortality in this age group. Further evaluation by probable cause of death revealed ‘referral’ as the leading COMCAT for acute respiratory infections and diarrhoeal disease, both of which accounted for large proportions of deaths in children, and in TB deaths, a leading cause of death overall. The validity of ‘referral’ in the refined COMCAT system was thus confirmed by the potential significance of referral challenges in the population. Figure 3 displays the sub-group analysis with the additional COMCAT output for the top 10 causes of death.

### Relevance

To support district health management and planning, data that can be operationalised into meaningful targets for intervention and that correspond with existing programme areas were seen as relevant. For example, ‘referral’ is a potentially significant factor contributing to child mortality where patients are not able to re-access the health system, establishing an impetus for action and investment to reduce deaths in this category.

Assigning deaths to a single circumstantial category was seen as useful for uptake and action. Health systems stakeholders shared insights that continually reporting problems as multifactorial does not provide solutions and can strongly reduce the urgency to address individual contributing factors. Simplified and focused problem identification was articulated a better approach to driving solutions and policy change: identifying a specific set of problems with clear solutions and then measuring impacts of interventions on those to help to drive a response.

The ability to capture deaths that occur outside facilities was also identified as of major importance. Provincial and district targets and indicators for maternal, neonatal, infant and child mortality relate to facility deaths only [50,51]. Otherwise, policy and planning documents contained several targets and priority areas corresponding with COMCATs on care seeking, health behaviours, patient experience, quality of care, transport and emergency medical services [50,51].

The ability to provide a comprehensive picture of trends across multiple diseases, conditions, and longitudinally, was identified as a useful feature. In addition, being able to identify policy and programme successes (as well as challenges) with the data was highlighted as beneficial. For example, improvements observed in under-5 and HIV/AIDS-related mortality were accompanied by reductions in COMCATs ‘recognition’ and ‘accessing care’. These are likely to reflect effective health information campaigns and removal of financial barriers.

**Figure 4.**
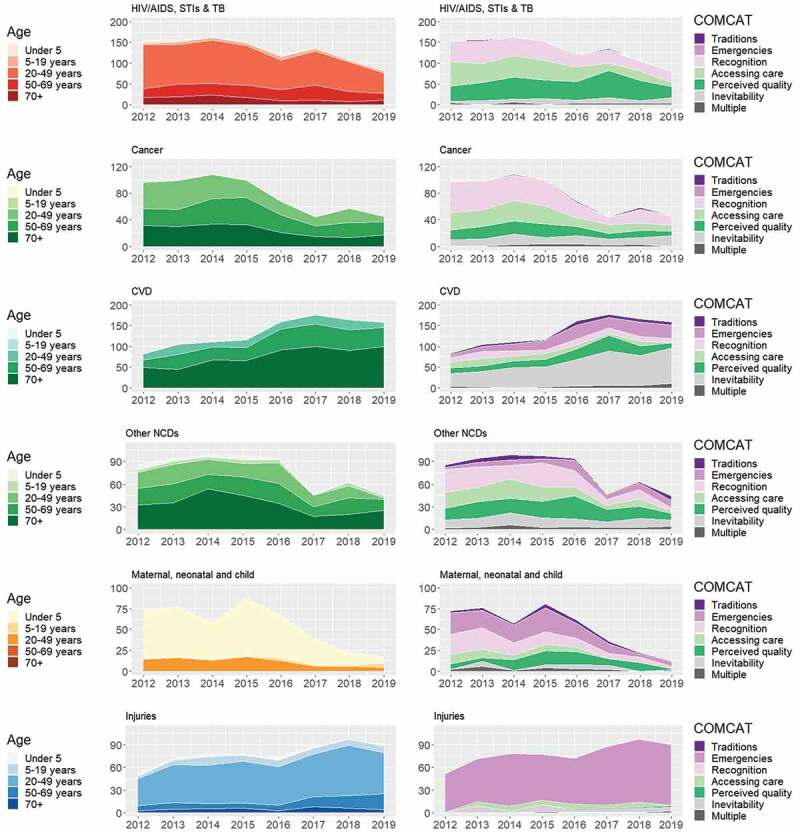
Number of deaths for health programme areas and circumstances of mortality categories (COMCATs) stratified by year and cause of death category for deaths in the Agincourt HDSS.

Otherwise, the importance of clear nomenclature was highlighted, along with the need for background information describing VA and how COMCATs are constructed. We produced different data presentations and visualisations arranging the COMCAT data by health priorities and programme areas, and developed accompanying explanatory notes (Figure 4, Supplementary Material 4, 5). We spent time balancing a sufficient level of granularity suitable for decision-making with that which could be reasonably achieved in large-scale population assessment.

Otherwise, VA and COMCAT were discussed as tools capable of making contributions in an overall toolbox of data and the greatest benefit is when different tools are used together in planning. Specifically, the District Health Information System (DHIS) (an open-source software platform used for reporting, analysis and dissemination of data in the public health sector [53]) provides a facility-based picture, while VA and COMCAT add a social and health systems dimension and provide an overall account inclusive of community deaths. Together, these tools are complementary. Finally, considering the relatively early stage of development, the need for continued engagement and exchange between researchers and health systems stakeholders to develop the COMCAT system and its use was recommended.

## Discussion

The COMCAT system reliably produced results that made sense across broadly similar settings and over time. Reductions in under-5 mortality, characterised by falling levels of infectious diseases and all COMCATs, can be interpreted in terms of high coverage of basic maternal, newborn and child health services within broader, integrated approaches to maternal and child wellbeing in South Africa [54]. Similarly, the decline in HIV/AIDS and TB mortality in the 20–49 years age group and reductions in COMCATs ‘recognition’, ‘accessing care’ and ‘perceived quality’ are consistent with increased uptake of accessible HIV and TB treatment [55].

Nevertheless, among 5–19 and 20–49 year olds, a substantial burden was attributed to injuries and the ‘emergencies’ COMCAT. While HIV/AIDS is declining in the country, drug and alcohol abuse is *increasing* among youth and adolescents linked to high levels of violence and road traffic accidents and emergency medical services of low quality [56,57]. NCDs predominated in the 50–69 and 70+ years age groups where most deaths were attributed to CVD and ‘emergencies’ and ‘inevitable’ COMCATs. This reflects an exponentially increasing NCD burden in South Africa and a lack of emergency and preventative health system responsiveness, which threatens achievements in infectious disease control and maternal and child health [58,59]. As in many settings, the authorities often have neither reliable data on the population burden nor its determinants, and thus are unable to plan and organize services responding to local needs, and particularly the needs of vulnerable and excluded groups.

We identified gaps in the COMCAT system and developed an additional category, ‘referral’, which accounted for a significant proportion of deaths in sub-group analysis. Poor referral is a key contributor to avoidable child deaths in South Africa and across sub-Saharan Africa [45,46]. In addition, delayed referral has been established as a leading social cause of adult mortality [48]. While it was not possible to incorporate home care recommendations directly, the refined COMCATs capture a more complete picture of critical limiting circumstances suitable for a range of conditions and settings.

We refined the COMCAT system with and for health systems stakeholders. While VA and COMCAT data do not entirely correspond with DHIS indicators, they are complementary. We developed data amenable to translation into action, visualisations by existing care models, and committed to continued exchange to build data from different sources to tell a common story. While these are useful developments, data and data use are not the norm in district health systems. DHISs are often costly, producing poor quality information with limited uptake in practice and policy development [60–65].

Exacerbating these problems, researchers often neither align to local contexts nor engage and maintain relationships with policy and systems stakeholders [65]. Global health research in particular is notorious for extractive and decontextualised practices, disenfranchising countries from their own data [66,67]. Research in HDSSs is seen to focus on ‘international value’ at the expense of contributing to national and sub-national policy priorities, and uptake of and support for external research by the authorities [68].

Embedding VA tools in health and civil registration is thus proposed a long-term strategy to support ‘learning health systems’ with locally relevant data based on core standards and cognisant of the social and health systems drivers of avoidable mortality [69]. We have previously documented VA studies engaging in service and emergency responses to close critical information gaps [70,71]. Moreover, COVID-19 underscores the necessity of real-time local data [7,72,73].

We focussed on refining the tool with and for district health planning. Routine use must also, however, include CRVS systems for continuous, permanent, compulsory and universal mortality and cause of death data. There is significant potential for VA and COMCAT to assist CRVS, and DHIS, to provide a more complete statistical picture for countries. Acknowledging CRVS, DHIS and HDSS are complex systems, consolidating expertise, building common purpose, strategic collaborations and capacities addressing organisational, technical, and behavioural challenges are priorities [9,60]. As stable public health observatories, HDSSs occupy strategically important positions between authorities and communities [57,68]. Considering who VA aims to represent, community actors should be seen as critical stakeholders to improve notification of community deaths: ‘considering these are the very communities systematically missed by routine information systems’’ [68]. Cooperative working to understand mortality in local contexts and local terms, and in terms of who uses which data and how, are key areas for future development [74–76].

### Strengths and limitations

The COMCAT system is based on extensive literature reviews and a focus on caregiver perspectives on health system responsiveness at and around time of death [21–23]. The COMCAT system is embedded in international standard classification systems as a long-term strategy for more relevant and robust local data. The system is flexible: it can be employed for specific conditions and programmes and as a planning tool, providing a comprehensive picture of health situations and trends across multiple diseases and conditions and longitudinally. The tool is primarily aimed at service planners and managers to guide priority-setting; however, it can also be used to monitor progress towards national and international goals by aggregating locally produced data [77]. COMCATs appear to reveal sex-differences to a greater extent than medical causes. Indeterminate medical causes, which accounted for 13.5% of all deaths in the dataset, can also potentially be elucidated by COMCATs.

COMCAT and VA, however, remain imperfect tools to understand complex realities. While depicted sequentially by pathways models, seeking and receiving care are often non-linear processes with care sought multiple times from multiple providers [78,79]. To capture key critical, limiting circumstances and events, the COMCAT model selects the one, most probable, category as an initial, simple, reproducible attempt to broaden ways to understand avoidable and unregistered deaths. We minimised overlap and ambiguity between circumstantial categories in the model and assigned a ‘multiple’ category when no one COMCAT dominates. To support uptake, health systems stakeholders advised that attributing deaths to the ‘multiple’ COMCAT is unhelpful and it is more favourable to the end user to assign a specific COMCAT and measure the impact of interventions on single COMCATs.

We acknowledge that this approach may oversimplify situations of multidimensional hardship and entrenched health systems challenges and focus attention for intervention in potentially reductive ways. Interactions between constructs are thus acknowledged and further refinement should explore alternative ways to express multiple circumstantial drivers. For example, the approach used with InterVA is to split deaths proportionally between multiple medical causes, according to their probabilities, which are then aggregated across all deaths to give population cause-specific mortality fractions. Moreover, capturing wider social conditions of death (e.g. occupational and environmental) and how these interact with circumstances and events at and around time of death is a priority [80].

In terms of data included, HDSSs exhaustively cover district-level populations in deprived rural, peri-urban or urban areas and data extensively conform with other estimates, supporting their generalisability [footnote] [81]. We were unable to conduct analyses for the AHRI HDSS for the period 2012–2016, however, as these indicators were only included in their VA instrument since 2017. There was also a possibility that some stillbirths, neonatal and infant deaths may not be recorded [82]. This has been minimised in recent years. Since 2000, the most recent child born to each woman is named on the pre-populated household roster with careful enquiry around pregnancies or births since then and, since 2006, there has been careful probing about the pregnancy status of every woman of childbearing age [83]. Otherwise, assessment of circumstantial inputs was based on reasonable ranges, however different results may have been observed using interquartile ranges, for example.

Finally, reflecting on our positionality, the research team was predominantly based at or affiliated with HDSSs or Mpumalanga Department of Health, with some members located in the UK. As such, the team possessed a broad appreciation of data-driven decision-making across diverse contexts, unified by commitments to more distributed forms of evidence-informed decision-making supporting improvements in services and outcomes in rural populations.

## Conclusions

Official mortality data often do not represent neglected populations and, despite knowledge on the content and efficacy of many life-saving packages of care, evidence on their implementation is lacking. The COMCAT system is a pragmatic and scalable approach providing basic information, not available from other sources, on care seeking and utilisation at and around time of death in connection with medical outcomes for all deaths in populations.

We refined the COMCATs for inclusion in standard interpretation and mortality classification systems. We appraised reliability: highlighted key COMCATs contributing to avoidable mortality by age, disease and over time. We strengthened validity: identified referral as a critical gap, developed and tested appropriate indicators for use in future. Finally we developed recommendations to support uptake and future development.

The refined COMCATs capture a more complete picture of critical limiting circumstances suitable for a range of conditions and settings, enhancing the functionality of standard VA to promote analysis of circumstances of death as a routine analytic component in addition to medical cause. The developments focussed on routine use of VA and COMCAT in district health systems, acknowledging the need for development together with CRVS systems and community stakeholders, and that researchers and research infrastructure can broker and strengthen alliances and capacities for data collection, analysis and use.

As a method transitioning towards more distributed application, VA is well placed to support countries and communities to generate information on their own situations, and use this information to advance bottom-up learning and action. Continued development among a range of stakeholders will strengthen strategic alliances, as well as the ability of the tool to capture social and health systems drivers of avoidable mortality, and promote use in practice settings. There is great potential to support virtuous cycles of representation, data, action and learning from action on preventable community deaths on which little is otherwise known.

## Supplementary Material

Supplemental Material

## Data Availability

Data are available from the respective HDSS repositories.

## Paper context

Social determinants are recognised as the fundamental causes of avoid-able mortality. Systematic, consistent and scalable attribution of circumstantial categorisations of deaths has not been developed or used, however. The COMCAT system enhances functionality of VA to promote analysis of circumstances as a routine analytic component in addition to medical cause for service organisation and delivery. InterVA-5 is the first model that generates COMCAT output in VA processing, in parallel to independently generating probable medical causes of death.

## *‘A few authors have argued that the political legitimacy and technical validity of global health estimates would be improved if estimation processes worked from the bottom up’* (49)

Peter was one of the few. His work addressed a grand *moral and empirical* challenge: ‘the unequal world of heath data’. Through his mentorship, he encouraged and supported colleagues across the board to embrace difficult questions around material and data poverty. Peter was a unique role model. He led by example through long-standing, far-reaching, critical contributions to building health information systems and capacity internationally in health and sociodemographic surveillance systems (HDSS) processes and infrastructure in LMICs. All of it to understand preventable, ‘invisible’ mortality at scale.

Embedded in HDSS settings in the INDEPTH Network, an international network of HDSSs, we collaborated from the early 2000s to extend verbal autopsy (VA) to complement data on biomedical causes with information on ‘social causes of death’. This work combined data on how deaths occur (in terms of biomedical processes like organ failure and blood loss), with information on why they happened (in terms of circumstantial drivers and root causes), combining numbers representing burden of disease with information on the human experience of that burden. The work culminated in an extension to the international VA standard in 2012, updating it to routinely collect information on critical limiting circumstances and events at and around the time of death.

Our collaboration endured. We progressed ideas about how to further extend and, critically, *use information* on bio-social causes of death in new partnerships in South Africa with Mpumalanga Department of Health. Together with a founding member of the INDEPTH Network, the MRC/Wits-Agincourt HDSS, we built multi-sectoral collaborations between government and non-government agencies, researchers and rural communities to coproduce evidence including with VA on local health concerns, and act on these data in cooperative, multi-agency partnerships (www.vapar.org). This has been a major contribution to realising the potential of the method outside research settings, in policy, planning and practice.

Peter had a profound influence on this research, which has included the training of many postgraduates who have gone onto build careers in HDSS and elsewhere in global public health. Peter taught us not only the importance of combining different methodological perspectives, but also that good science is working collaboratively and inclusively, with decency and integrity, to have faith and to be fearless! We are deeply privileged to have worked with Peter, he was internationally renowned for his work measuring global burden of disease and was a strong advocate of cooperation in science. Peter was a mentor and colleague to so many of us in the global health community, a true gentleman, dear friend and brilliant scholar. He will be deeply missed.

## FOOTNOTE From source (68)

HDSS bring additional data to bear: ‘all-cause mortality rates, cause-specific mortality fractions and rates, and life table probabilities, and fertility and migration rates. Key outputs include population and household characteristics (including social-economic status), disease burden, use of health services, and exposure to environmental risk factors. The associated data on environment, education, immunisation status, housing, access to water, sanitation, and energy provide deep explanatory contexts to the observed dynamics in health and demographic outcomes. In an HDSS, an initial baseline census is conducted of all households and residents in the defined geographic area; data is updated subsequently several times a year through enumeration update rounds. Data can fully complement the Health Management Information System through population-based data that is independent of the use of healthcare services, and can therefore be used for health planning.’

## References

[1] Setel PW, Macfarlane SB, Szreter S, et al. A scandal of invisibility: making everyone count by counting everyone. Lancet. cited 2018 Sep 29 2007;Nov 370:1–15. Available from. 17992727 10.1016/S0140-6736(07)61307-5

[2] Horton R Counting for health. Lancet. 2007 Nov [cited 2018 Sep 29];370:1526. Available from:17992726 10.1016/S0140-6736(07)61418-4

[3] Marinda E, Simbayi L, Zuma K, et al. Towards achieving the 90–90–90 HIV targets: results from the South African 2017 national HIV survey. BMC Public Health. 2020;20:1375. 32907565 10.1186/s12889-020-09457-z PMC7487872

[4] Basera TJ, Schmitz K, Price J, et al. Community surveillance and response to maternal and child deaths in low- and middle-income countries: a scoping review. PLoS One. 2021 Mar 16 [cited 2021 Mar 25];16:e0248143. Available from: https://dx.plos.org/10.1371/journal.pone.0248143 33725013 PMC7963102

[5] Byass P. The unequal world of health data. PLoS Med [Internet]. 2009 Nov 24 [cited 2021 Mar 2];6 e1000155:. Available from: https://dx.plos.org/10.1371/journal.pmed.1000155 PMC2777404 19956675

[6] Sankoh O. Why population-based data are crucial to achieving the sustainable development goals on behalf of the INDEPTH network and partners ¥. Int J Epidemiol [Internet]. 2017 [cited 2021 Mar 22];46:4–7. Available from: www.un.org/sustainabledevelopment/sustainable-development-10.1093/ije/dyx010 PMC5837246 28204483

[7] Setel P, Abouzahr C, Atuheire EB, et al. Mortality surveillance during the COVID-19 pandemic [Internet]. Bull World Health Organ World Health Organization; 2020 [cited 2021 Mar 22]. p. 374. Available from. ; 98: . 32514207 10.2471/BLT.20.263194 PMC7265935

[8] Fottrell E, Byass P Verbal autopsy: methods in transition. Epidemiol Rev. 2010;32:38–55. 20203105 10.1093/epirev/mxq003

[9] Lopez AD, McLaughlin D, Richards N Reducing ignorance about who dies of what: research and innovation to strengthen CRVS systems. BMC Med 2020 181 [Internet]. 2020 Mar 9 [cited 2021 Oct 6];18:1–6. Available from: https://bmcmedicine.biomedcentral.com/articles/10.1186/s12916-020-01526-9 PMC7061482 32146906

[10] Byass P, Huong DL, Van Minh H A probabilistic approach to interpreting verbal autopsies: methodology and preliminary validation in Vietnam. Scand J Public Health [Internet]. 2003 Dec 7 [cited 2021 Mar 2];31:32–37. Available from: http://journals.sagepub.com/doi/10.1080/14034950310015086 14649636

[11] Byass P, Fottrell E, Huong DL, et al. Refining a probabilistic model for interpreting verbal autopsy data. Scand J Public Health [Internet]. 2006 Jan 5 [cited 2021 Mar 2];34:26–31. Available from: http://journals.sagepub.com/doi/10.1080/14034940510032202 PMC2833983 16449041

[12] Byass P, Chandramohan D, Clark SJ, et al. Strengthening standardised interpretation of verbal autopsy data: the new InterVA-4 tool. Glob Health Action. 2012; 5: 19281. 10.3402/gha.v5i0.19281 PMC3433652 22944365

[13] Byass P, Hussain-Alkhateeb L, D’Ambruoso L, et al. An integrated approach to processing WHO-2016 verbal autopsy data: the InterVA-5 model. BMC Med [Internet]. 2019 May 30 [cited 2021 Mar 2];17:102. Available from: https://bmcmedicine.biomedcentral.com/articles/10.1186/s12916-019-1333-6 PMC6543589 31146736

[14] Bird J, Byass P, Kahn K, et al. A matter of life and death: practical and ethical constraints in the development of a mobile verbal autopsy tool. In: Conference on Human Factors in Computing Systems - Proceedings [Internet]. New York, NY, USA: ACM; 2013 [cited 2021 Mar 2]. p. 1489–1498. Available from: https://dl.acm.org/doi/10.1145/2470654.2466198

[15] WHO. Verbal autopsy standards: the 2012 WHO verbal autopsy instrument release candidate 1 [Internet]. Geneva: World Health Organization, cited 2021 Mar 2]. Available from https://www.who.int/healthinfo/statistics/WHO_VA_2012_RC1_Instrument.pdf

[16] Paxton A, Bailey P, Lobis S The United Nations process indicators for emergency obstetric care: reflections based on a decade of experience. Int J Gynecol Obstet. 2006;95:192–208. 10.1016/j.ijgo.2006.08.009 17074557

[17] WHO. The stop TB strategy: building on and enhancing DOTS to meet the TB-related millennium development goals [Internet]. Geneva: WHO; 2006. [cited 2021 Apr 20]. Available from https://apps.who.int/iris/bitstream/handle/10665/69241/WHO_HTM_STB_2006.368_eng.pdf?sequence=1&isAllowed=y

[18] WHO. Beyond the Numbers Reviewing maternal deaths and complications to make pregnancy safer. Geneva: WHO; 2004. 10.1093/bmb/ldg009 14711752

[19] Koffi AK, Kalter HD, Loveth EN, et al. Beyond causes of death: the social determinants of mortality among children aged 1-59 months in Nigeria from 2009 to 2013. PLoS One. 2017 May 1 [cited 2021 Mar 2];12. Available from: https://pubmed.ncbi.nlm.nih.gov/28562610/10.1371/journal.pone.0177025 PMC5451019 28562610

[20] D’Ambruoso L, Byass P, Qomariyah SN, et al. A lost cause? Extending verbal autopsy to investigate biomedical and socio-cultural causes of maternal death in Burkina Faso and Indonesia. Soc Sci Med. 2010;71:1728–1738. 20646807 10.1016/j.socscimed.2010.05.023

[21] Hussain-Alkhateeb L, D’Ambruoso L, Tollman S, et al. Enhancing the value of mortality data for health systems: adding Circumstances Of Mortality CATegories (COMCATs) to deaths investigated by verbal autopsy. Glob Health Action [Internet]. 2019 Jan 1 [cited 2019 Oct 26];12:1680068. Available from: https://www.tandfonline.com/doi/full/10.1080/16549716.2019.1680068 PMC6818104 31648624

[22] D’Ambruoso L Care in obstetric emergencies : quality of care, access to care and participation in health in rural Indonesia. PhD Thesis University of Aberdeen [Internet]. [Aberdeen]: University of Aberdeen; 2011 [cited 2021 Apr 29]. Available from: https://abdn.primo.exlibrisgroup.com/discovery/fulldisplay?docid=alma990014073470205941&context=L&vid=44ABE_INST:44ABE_VU1&lang=en&search_scope=MyInst_and_CI&adaptor=LocalSearchEngine&tab=Everything&query=any,contains,careinobstetricemergencieslucia

[23] D’Ambruoso L, Kahn K, Wagner RG, et al. Moving from medical to health systems classifications of deaths: extending verbal autopsy to collect information on the circumstances of mortality. Glob Heal Res Policy. 2016;1:2. 10.1186/s41256-016-0002-y PMC5675065 29202052

[24] Edem IJ, Dare AJ, Byass P, et al. External injuries, trauma and avoidable deaths in Agincourt, South Africa: a retrospective observational and qualitative study. BMJ Open. 2019 Jun 1 [cited 2021 Mar 23]; 9: 27576. Available from http://bmjopen.bmj.com/10.1136/bmjopen-2018-027576 PMC6561452 31167869

[25] Fraser A, Newberry Le Vay J, Byass P, et al. Time-critical conditions: assessment of burden and access to care using verbal autopsy in Agincourt, South Africa. BMJ Glob Heal [Internet]. 2020 Apr 16 [cited 2021 Mar 23];5:2289. Available from: http://gh.bmj.com/10.1136/bmjgh-2020-002289 PMC7199706 32377406

[26] Bradshaw D, Joubert J, Maqungo M, et al. South African national cause-of-death validation project: methodology and description of a national sample of verbal autopsies [Internet]. Cape Town:South African Medical Research Council. 2020 cited 2021 Apr 14 Available from https://www.samrc.ac.za/sites/default/files/files/2021-02-04/NationalCause-of-deathValidationReport.pdf

[27] Statistics South Africa. Mid-year population estimates 2020. statistical release P0302 [Internet]. Pretoria: Statistics South Africa; 2021. [cited 2021 May 10]Available from http://www.statssa.gov.za/publications/P0302/P03022020.pdf

[28] Neethling I, Groenewald P, Schneider H, et al. Trends and inequities in amenable mortality between 1997 and 2012 in South Africa. S Afr Med J. 2019;109:597–604. 31456556 10.7196/SAMJ.2019.v109i8.13796

[29] Shisana O, Rehle T, Simbayi L, et al. South African national HIV prevalence, incidence and behaviour survey. HSRC; Cape Town. 2012. 2014.

[30] Bhalla K, Harrison J, Shahraz S, et al. Burden of road injuries in sub-Saharan Africa. Baltimore: Johns Hopkins Bloomberg School of Public Health; 2014.

[31] Government of the Republic of South Africa. The reconstruction and development programme (RDP): a policy framework. Pretoria: Government of the Republic of South Africa; 1994.

[32] Government of the Republic of South Africa. national health act [Internet]. Pretoria: Government of the Republic of South Africa; 2003. [cited 2018 Sep 29]. Available from https://www.gov.za/documents/national-health-act

[33] Coovadia H, Jewkes R, Barron P, et al. The health and health system of South Africa: historical roots of current public health challenges. Lancet. 2009;374:817–834. 19709728 10.1016/S0140-6736(09)60951-X

[34] Kahn K, Collinson MA, Gomez-Olive FX, et al. Profile: agincourt health and socio-demographic surveillance system. Int J Epidemiol. 2012;41:988–1001. 22933647 10.1093/ije/dys115 PMC3429877

[35] Collinson M, Herbst K, Tollman S, et al. South Africa - SAPRIN Individual Surveillance Episodes 2020 [Internet]. SAPRIN.SIS. SAPRIN; 2020. p. 1–26. Available from: http://saprindata.samrc.ac.za/index.php/catalog/29

[36] Tanser F, Hosegood V, Bärnighausen T, et al. Cohort profile: africa centre demographic information system (ACDIS) and population-based HIV survey. Int J Epidemiol [Internet]. 2008 [cited 2021 May 1]; 37:956–962.Available from 10.1093/ije/dym211 PMC2557060 17998242

[37] Siedner MJ, Harling G, Derache A, et al. Protocol: leveraging a demographic and health surveillance system for Covid-19 Surveillance in rural KwaZulu-Natal. Wellcome Open Res Internet]. 2020 Aug 25 [cited 2021 May 1];5:.Available from: :109. 32802963 10.12688/wellcomeopenres.15949.1 PMC7424917

[38] Harling G, Gómez-Olivé FX, Tlouyamma J, et al. Protective behaviours and secondary harms from non-pharmaceutical interventions during the COVID-19 epidemic in South Africa: a multisite prospective longitudinal study (Preprint). JMIR Public Heal Surveill [Internet]. 2020 Nov 26 [cited 2021 May 14];7:e26073. Available from:10.2196/26073 PMC8121138 33827046

[39] Gareta D, Baisley K, Mngomezulu T, et al. Cohort profile update: africa centre demographic Information System (ACDIS) and population-based HIV survey. Int J Epidemiol [Internet]. 2021 Feb 1 [cited 2021 Oct 6];50:33. Available from:: /pmc/articles/PMC7938501/10.1093/ije/dyaa264 PMC7938501 33437994

[40] Cowan E, D’Ambruoso L, Price J, et al. Dataset: a consolidated and harmonised verbal autopsy dataset from health and demographic surveillance sites in South Africa. F1000Res. 2021. 10.12688/f1000research.55377.1 PMC11399759 39282516

[41] Byass P, Hussain-Alkhateeb L, D’Ambruoso L, et al. An integrated approach to processing WHO-2016 verbal autopsy data: the InterVA-5 model. BMC Med. 2019 May 30;17(1). 31146736 10.1186/s12916-019-1333-6 PMC6543589

[42] InterVA - software for verbal autopsy [Internet]. [cited 2021 Mar 22]. Available from: http://www.byass.uk/interva/

[43] Waldman R, Campbell CC, Steketee RW. Overcoming remaining barriers: the pathway to survival [Internet]. Arlington: BASICS Basic Support for Institutionalizing Child Survival; 1996. cited 2021 May 1. Available from https://pdf.usaid.gov/pdf_docs/PNABZ644.pdf

[44] Koffi AK, Maina A, Yaroh AG, et al. Social determinants of child mortality in Niger: results from the 2012 national verbal and social autopsy study. J Glob Health [Internet]. 2016 [cited 2021 Mar 2];6. Available from: /pmc/articles/PMC4766790/10.7189/jogh.06.010603 PMC4766790 26955473

[45] Price J, Willcox M, Kabudula CW, et al. Care pathways during a child’s final illness in rural South Africa: findings from a social autopsy study. PLoS One. 2019 Oct 22 [cited 2021 Mar 2];14:e0224284. Available from: https://dx.plos.org/10.1371/journal.pone.0224284 31639177 PMC6804973

[46] Price J, Lee J, Willcox M, et al. Place of death, care-seeking and care pathway progression in the final illnesses of children under five years of age in sub-Saharan Africa: a systematic review. J Glob Health [Internet]. 2019 [cited 2021 Apr 18];9. Available from: /pmc/articles/PMC6815655/10.7189/jogh.09.020422 PMC6815655 31673338

[47] Cheryl AM, Cassidy J, Elizabeth K et al. Using social autopsy to understand maternal, newborn, and child mortality in low-resource settings: a systematic review of the literature Glob Health Action.2017;10(1):1413917. doi:10.1080/16549716.2017.1413917 cited 2021 May 1 PMC5757230 29261449

[48] Gupta M, Kaur M, Lakshmi PVM, et al. Social autopsy for identifying causes of adult mortality. PLoS One. 2018 May 31 [cited 2021 May 1];13:e0198172. Available from: https://dx.plos.org/10.1371/journal.pone.0198172 29851982 PMC5978887

[49] Pisani E, Kok M In the eye of the beholder: to make global health estimates useful, make them more socially robust. Glob Health Action [Internet]. 2017 [cited 2021 Apr 15];10. Available from: 1266180 10.3402/gha.v9.32298 PMC5124117 28532303

[50] Mpumalanga Department of Health. Annual Performance Plan (APP) 2020-21. Mbombela: Mpumalanga Department of Heatlh; 2020.

[51] Mpumalanga Department of Health. Ehlanzeni District Municipality (DC32) District Health Plan 2020/21-2022/23 Mpumalanga Province. Mbombela: Mpumalanga Department of Health; 2020.

[52] Kalter HD, Salgado R, Babille M, et al. Social autopsy for maternal and child deaths: A comprehensive literature review to examine the concept and the development of the method [Internet]. Vol. 9, Popul Health Metr. 2011 [cited 2021 Mar 2]. Available from: 10.1186/1478-7954-9-45 PMC3160938 21819605

[53] University of Oslo. DHIS2 [Internet]. [cited 2021 May 10]. Available from: https://dhis2.org/

[54] Shung-King M, Lake L, Sanders D, et al. South African Child Gauge 2019. Child and adolescent health: leave no one behind [Internet]. Cape Town: Children’s Institute, University of Cape Town; 2019. cited 2021 May 14. Available from http://www.ci.uct.ac.za/sites/default/files/image_tool/images/367/Child_Gauge/South_African_Child_Gauge_2019/ChildGauge_2019_final_print%28sm%29.pdf

[55] Meyer-Rath G, Johnson LF, Pillay Y, et al. Changing the South African national antiretroviral therapy guidelines: the role of cost modelling PLoS One 2017 Oct 1 cited 2021 May 14 12 Available from:/pmc/articles/PMC5662079/10.1371/journal.pone.0186557 PMC5662079 29084275

[56] Department of Health. National adolescent and youth health policy 2017 [Internet]. Pretoria: Department of Health; 2017. Available from: https://tinyurl.com/y5wbkpw8

[57] D’Ambruoso L, Twine R, Mabetha D, et al. ‘Voice needs teeth to have bite’! Community-led multisectoral action-learning to address alcohol and drug abuse in rural South Africa. Unpublished manuscript. 10.1371/journal.pgph.0000323 PMC10022044 36962488

[58] Howard I, Cameron P, Wallis L, et al. Understanding quality systems in the South African prehospital emergency medical services: a multiple exploratory case study. BMJ Open Qual Internet]. 2020 [cited 2021 May 14];9:946. Available from. 10.1136/bmjoq-2020-000946 PMC7247383 32439739

[59] Pillay-van Wyk V, Msemburi W, Laubscher R, et al. Mortality trends and differentials in South Africa from 1997 to 2012: second national burden of disease study. Lancet Glob Heal [Internet]. 2016 Sep 1 [cited 2019 Jul 13];4:e642–53. Available from:10.1016/S2214-109X(16)30113-9 27539806

[60] WHO. WHO civil registration and vital statistics strategic Implementation Plan 2021-2025 [Internet].World Health Organization:Geneva 2021.Available from https://getinthepicture.org/sites/default/files/resources/WHOCRVSstrategicimplementationplan.pdf

[61] Garrib A, Stoops N, McKenzie A, et al. An evaluation of the district health information system in rural South Africa. S Afr Med J. 2008;98:549–552. 18785397

[62] Hotchkiss DR, Aqil A, Lippeveld T, et al. Evaluation of the performance of routine information system management (PRISM) framework: evidence from Uganda. BMC Health Serv Res [Internet]. 2010 [cited 2021 Apr 14];10:188. Available from: http://www.biomedcentral.com/1472-6963/10/188 10.1186/1472-6963-10-188 PMC2904760 20598151

[63] Rouamba T, Samadoulougou S, Kirakoya-Samadoulougou F Addressing challenges in routine health data reporting in Burkina Faso through Bayesian spatiotemporal prediction of weekly clinical malaria incidence. Sci Rep [Internet]. 2020 Dec 1 [cited 2021 Mar 7];10. Available from: https://pubmed.ncbi.nlm.nih.gov/33024162/10.1038/s41598-020-73601-3 PMC7538437 33024162

[64] Nshimyiryo A, Kirk CM, Sauer SM, et al. Health management information system (HMIS) data verification: a case study in four districts in Rwanda. PLoS One Internet]. 2020 Jul 1 [cited 2021 Mar 7];. Available from. ;15:e0235823. 32678851 10.1371/journal.pone.0235823 PMC7367468

[65] Sigudla J A model to facilitate research uptake in health care practice and policy development for low-resourced countries. Unpublished manuscript.

[66] Horton, R. Offline:. The case against global health. Lancet 2014;383. 10.1016/S0140-6736(14)60797-2. 37210107

[67] Reidpath DD, Allotey P The problem of “trickle-down science” from the global north to the global South [Internet]. Vol. 4, BMJ Glob Health. BMJ Publishing Group; 2019 [cited 2021 Apr 29]. p. 1719. Available from:10.1136/bmjgh-2019-001719 PMC6666820 31406597

[68] De Savigny D, Renggli S, Cobos D, et al. Maximizing synergies between health observatories and CRVS: guidance for INDEPTH HDSS Sites and CRVS Stakeholders [Internet]. INDEPTH Network and the Bloomberg Data for Health Initiative; 2018 [cited 2021 Mar 20]. Available from: https://getinthepicture.org/sites/default/files/resources/MaximizingSynergiesbetweenHealthObservatoriesandCRVSv2.5.pdf

[69] Sheikh K, Agyepong I, Jhalani M, et al. Learning health systems: an empowering agenda for low-income and middle-income countries [Internet]. 395, The Lancet. Lancet Publishing Group; 2020 [cited 2021 Apr 20]. p. 476–477. Available from https://pubmed.ncbi.nlm.nih.gov/32061280/10.1016/S0140-6736(19)33134-4 32061280

[70] Thomas LM, D’Ambruoso L, Balabanova D Verbal autopsy in health policy and systems: a literature review [Internet]. Vol. 3, BMJ Glob Health. BMJ Publishing Group; 2018 [cited 2021 Apr 29]. p. 639. Available from:10.1136/bmjgh-2017-000639 PMC5935163 29736271

[71] Thomas LM, D’Ambruoso L, Balabanova D Use of verbal autopsy and social autopsy in humanitarian crises. BMJ Glob Heal [Internet]. 2018 Nov 1 [cited 2021 May 12;3:640. Available from: http://gh.bmj.com/10.1136/bmjgh-2017-000640 PMC5935165 29736275

[72] Loewenson R, Colvin CJ, Szabzon F, et al. Beyond command and control: a rapid review of meaningful community-engaged responses to COVID-19. Glob Public Health [Internet]. 2021 Mar 18 [cited 2021 Mar 21];1–15. Available from: http://www.ncbi.nlm.nih.gov/pubmed/33734007 10.1080/17441692.2021.1900316 33734007

[73] Greenhalgh GT Will COVID-19 be evidence-based medicine’s nemesis? 2020;PLOS Med. 17:e1003266. 32603323 10.1371/journal.pmed.1003266 PMC7326185

[74] Abimbola S The uses of knowledge in global health. BMJ Glob Heal [Internet]. 2021 [cited 2021 Apr 18];6: Available from https://gh.bmj.com/content/bmjgh/6/4/e005802.full.pdf 10.1136/bmjgh-2021-005802 PMC8030475 33820807

[75] Houle B, Angotti N, Clark SJ, et al. Let’s talk about sex, maybe: interviewers, respondents, and sexual behavior reporting in rural South Africa. Field Methods. 2016;28:112–132. 28190977 10.1177/1525822X15595343 PMC5300069

[76] Gouda HN, Flaxman AD, Brolan CE, et al. New challenges for verbal autopsy: considering the ethical and social implications of verbal autopsy methods in routine health information systems [Internet]. Soc Sci Med Elsevier Ltd; 2017 [cited 2021 Apr 29]. p. 65–74. Available from. ; 184:28501755 10.1016/j.socscimed.2017.05.002

[77] AbouZahr C, Boerma T, Hogan D Global estimates of country health indicators: useful, unnecessary, inevitable?Vol. , Global Health Action. 2017;10(S1):1290370. [cited 2021]. 10.1080/16549716.2017.1290370 PMC5645718 28532307

[78] Sharkey AB, Chopra M, Jackson D, et al. Pathways of care-seeking during fatal infant illnesses in under-resourced South African settings. Trans R Soc Trop Med Hyg [Internet]. 2012 Feb 1 [cited 2021 Apr 27];106:110–116. Available from: https://academic.oup.com/trstmh/article-lookup/doi/10.1016/j.trstmh.2011.10.008 PMC3254810 22136954

[79] D’Ambruoso L, Byass P, Qomariyah SN Can the right to health inform public health planning in developing countries? A case study for maternal healthcare from Indonesia. Glob Health Action. 2008;1:1. 10.3402/gha.v1i0.1828 PMC2779912 20027244

[80] Lyatuu I, Winkler MS, Loss G, et al. Estimating the mortality burden of large scale mining projects—Evidence from a prospective mortality surveillance study in Tanzania. Chirico F, editor. PLOS Glob Public Heal [Internet]. 2021 Oct 13 [cited 2021 Oct 21];1:e0000008. Available from: https://journals.plos.org/globalpublichealth/article?id= 10.1371/journal.pgph.0000008 PMC10021452 36962075

[81] Bocquier P, Sankoh O, Byass P Are health and demographic surveillance system estimates sufficiently generalisable? Glob Health Action [Internet]. 2017 Jan 18 [cited 2018 Jun 16];10:1356621. Available from:10.1080/16549716.2017.1356621 PMC5645714 28820344

[82] Waiswa P, Akuze J, Moyer C, et al. Status of birth and pregnancy outcome capture in health demographic surveillance sites in 13 countries. Int J Public Health [Internet]. [cited 2021 Apr 19];64. Available from: 10.1007/s00038-019-01241-0 PMC6614155 31240333

[83] Kabudula CW, Houle B, Collinson MA, et al. Progression of the epidemiological transition in a rural South African setting: findings from population surveillance in Agincourt, 1993-2013. BMC Public Health [Internet]. 2017 May 10 [cited 2021 Apr 29]; 17: 424. Available from http://bmcpublichealth.biomedcentral.com/articles/10.1186/s12889-017-4312-x PMC5424387 28486934

