# Supplementary material for: Refining circumstances of mortality categories (COMCAT): a verbal autopsy model connecting circumstances of deaths with outcomes for public health decision-making
Source: Glob Health Action. 2022 Apr 4;14(Suppl):2000091. doi: 10.1080/16549716.2021.2000091 (PMC8986216; doi:10.1080/16549716.2021.2000091)
Supplement: Supplemental Material [file ZGHA_A_2000091_SM0233.zip › z SM 1_CSMF and COMCAT R1.docx]

Supplementary Material 1

a: Causes of deaths in the Agincourt and AHRI HDSSs 2012-19 as assigned by InterVA-5 to verbal autopsy data (n=7980)

| Cause of death category | Agincourt |  | AHRI |  | Total |  |
| --- | --- | --- | --- | --- | --- | --- |
|  | N | % | N | % | N | % |
| HIV/AIDS + TB | 1065.44 | 17.99 | 537.65 | 26.15 | 1603.09 | 20.09 |
| Other Infections | 941.64 | 15.90 | 81.22 | 3.95 | 1022.86 | 12.82 |
| Cancer | 623.18 | 10.52 | 164.72 | 8.01 | 787.90 | 9.87 |
| CVD | 1078.06 | 18.20 | 532.44 | 25.90 | 1610.5 | 20.18 |
| Other NCD | 624.26 | 10.54 | 182.13 | 8.86 | 806.39 | 10.11 |
| Injuries | 627.77 | 10.60 | 291.31 | 14.17 | 919.08 | 11.52 |
| Neonatal | 18.38 | 0.31 | 15.57 | 0.76 | 33.95 | 0.43 |
| Maternal | 97.52 | 1.65 | 23.41 | 1.14 | 120.93 | 1.52 |
| Indeterminate | 847.75 | 14.31 | 227.55 | 11.07 | 1075.30 | 13.47 |
|  |  |  |  |  |  |  |
| Female | 2921 | 49.31 | 1025 | 49.85 | 3946 | 49.45 |
| Male | 3003 | 50.69 | 1031 | 20.15 | 4034 | 50.55 |
|  |  |  |  |  |  |  |
| Total | 5924 | 74.24 | 2056 | 25.76 | 7980 | 100 |

b: Circumstance of mortality categories (COMCATs) in the Agincourt and AHRI HDSSs 2012-19 as assigned by InterVA-5 to verbal autopsy data (n=7980)

| Circumstance of mortality categories (COMCATs) | Agincourt | | AHRI | | TOTAL | |
| --- | --- | --- | --- | --- | --- | --- |
|  | N | % | N | % | N | % |
| Traditions | 210 | 3.54 | 74 | 3.60 | 284 | 3.56 |
| Emergencies | 1160 | 19.58 | 440 | 21.40 | 1600 | 20.05 |
| Recognition | 1142 | 19.28 | 125 | 6.08 | 1267 | 15.88 |
| Accessing care | 1028 | 17.35 | 457 | 22.23 | 1485 | 18.61 |
| Perceived quality | 1145 | 19.33 | 280 | 13.62 | 1425 | 17.86 |
| Inevitable | 1005 | 16.96 | 610 | 29.67 | 1615 | 20.24 |
| Multiple | 234 | 3.95 | 70 | 3.40 | 304 | 3.81 |
|  |  |  |  |  |  |  |
| Female | 2921 | 49.31 | 1025 | 49.85 | 3946 | 49.45 |
| Male | 3003 | 50.69 | 1031 | 20.15 | 4034 | 50.55 |
|  |  |  |  |  |  |  |
| TOTAL | 5924 | 100 | 2056 | 100 | 7980 | 100 |

c: Circumstance of mortality categories (COMCATs) in the Agincourt and AHRI HDSSs 2012-19 as assigned by InterVA-5 to verbal autopsy data (n=7980), by cause of death category

| Circumstance of mortality categories (COMCATs) | HIV/TB | | Other infections | | Cancer | | CVD | | Other NCD | | Injuries | | Neonatal | | Maternal | | Indeterminate | | TOTAL | |
| --- | --- | --- | --- | --- | --- | --- | --- | --- | --- | --- | --- | --- | --- | --- | --- | --- | --- | --- | --- | --- |
|  | N | % | N | % | N | % | N | % | N | % | N | % | N | % | N | % | N | % | N | % |
| Traditions | 23.17 | 1.45 | 85.52 | 8.36 | 8.43 | 1.07 | 63.7 | 3.96 | 36.24 | 4.49 | 4.44 | 0.48 | 0.98 | 2.89 | 4.25 | 3.51 | 57.27 | 5.33 | 284 | 3.56 |
| Emergencies | 12.68 | 0.79 | 121.57 | 11.89 | 8.04 | 1.02 | 237.24 | 14.73 | 77.58 | 9.62 | 813.73 | 88.54 | 15.66 | 46.13 | 57.95 | 47.92 | 255.55 | 23.77 | 1600 | 20.05 |
| Recognition | 379.05 | 23.64 | 269.36 | 26.33 | 256.25 | 32.52 | 98.44 | 6.11 | 158.94 | 19.71 | 0.28 | 0.03 | 0.97 | 2.86 | 4.48 | 3.70 | 99.23 | 9.23 | 1267 | 15.88 |
| Accessing care | 457.49 | 28.54 | 192.44 | 18.81 | 214.03 | 27.16 | 154.89 | 9.62 | 190.73 | 23.65 | 39.54 | 4.30 |  | 0.00 | 18.71 | 15.47 | 217.17 | 20.20 | 1485 | 18.61 |
| Perceived quality | 537.1 | 33.50 | 227.81 | 22.27 | 129.72 | 16.46 | 187.59 | 11.65 | 166.79 | 20.68 | 6.08 | 0.66 | 6.63 | 19.53 | 27.68 | 22.89 | 135.6 | 12.61 | 1425 | 17.86 |
| Inevitability | 142.33 | 8.88 | 63.67 | 6.22 | 150.88 | 19.15 | 814.3 | 50.56 | 140.93 | 17.48 | 45.45 | 4.95 | 8.97 | 26.42 | 4.2 | 3.47 | 244.27 | 22.72 | 1615 | 20.24 |
| Multiple | 51.27 | 3.20 | 62.49 | 6.11 | 20.55 | 2.61 | 54.34 | 3.37 | 35.18 | 4.36 | 9.56 | 1.04 | 0.74 | 2.18 | 3.66 | 3.03 | 66.21 | 6.16 | 304 | 3.81 |
|  |  |  |  |  |  |  |  |  |  |  |  |  |  |  |  |  |  |  |  |  |
| Female | 786.42 | 49.06 | 473.43 | 46.28 | 408.6 | 51.86 | 979.22 | 60.80 | 417.31 | 51.75 | 212.16 | 23.08 | 10.99 | 32.37 | 120.93 | 100.0 | 536.94 | 49.93 | 3946 | 49.45 |
| Male | 816.67 | 50.94 | 549.43 | 53.72 | 379.3 | 48.14 | 631.28 | 39.20 | 389.08 | 48.25 | 706.92 | 76.92 | 22.96 | 67.63 | 0.00 | 0.00 | 538.36 | 50.07 | 4034 | 50.55 |
|  |  |  |  |  |  |  |  |  |  |  |  |  |  |  |  |  |  |  |  |  |
| TOTAL | 1603.09 | 20.09 | 1022.86 | 12.82 | 787.9 | 9.87 | 1610.5 | 20.18 | 806.39 | 10.11 | 919.08 | 11.52 | 33.95 | 0.43 | 120.93 | 1.52 | 1075.3 | 13.47 | 7980 | 100.00 |

d: Circumstance of mortality categories (COMCATs) in the Agincourt and AHRI HDSSs 2012-19 as assigned by InterVA-5 to verbal autopsy data (n=7980), by age group

| Circumstance of mortality categories (COMCATs) | Under 5 | | 5-19 | | 20-49 | | 50-69 | | 70+ | | Total | |
| --- | --- | --- | --- | --- | --- | --- | --- | --- | --- | --- | --- | --- |
|  | N | % | N | % | N | % | N | % | N | % | N | % |
| Traditions | 23 | 5.94 | 24 | 7.00 | 103 | 3.31 | 52 | 2.74 | 82 | 3.67 | 284 | 3.56 |
| Emergencies | 128 | 33.07 | 143 | 41.69 | 878 | 28.20 | 305 | 16.04 | 146 | 6.53 | 1600 | 20.05 |
| Recognition | 107 | 27.65 | 53 | 15.45 | 423 | 13.58 | 345 | 18.15 | 339 | 15.17 | 1267 | 15.88 |
| Accessing care | 31 | 8.01 | 54 | 15.74 | 703 | 22.58 | 378 | 19.88 | 319 | 14.27 | 1485 | 18.61 |
| Perceived quality | 62 | 16.02 | 49 | 14.29 | 737 | 23.67 | 292 | 15.36 | 285 | 12.75 | 1425 | 17.86 |
| Inevitability | 18 | 4.65 | 9 | 2.62 | 154 | 4.95 | 451 | 23.72 | 983 | 43.98 | 1615 | 20.24 |
| Multiple | 18 | 4.65 | 11 | 3.21 | 116 | 3.73 | 78 | 4.10 | 81 | 3.62 | 304 | 3.81 |
|  |  |  |  |  |  |  |  |  |  |  |  |  |
| Female | 172 | 44.44 | 144 | 41.98 | 1374 | 44.12 | 856 | 45.03 | 1400 | 62.64 | 3946 | 49.45 |
| Male | 215 | 55.56 | 199 | 58.02 | 1740 | 55.88 | 1045 | 54.97 | 835 | 37.36 | 4034 | 50.55 |
|  |  |  |  |  |  |  |  |  |  |  |  |  |
| TOTAL | 387 | 4.85 | 343 | 4.30 | 3114 | 39.02 | 1901 | 23.82 | 2235 | 28.01 | 7980 | 100.00 |


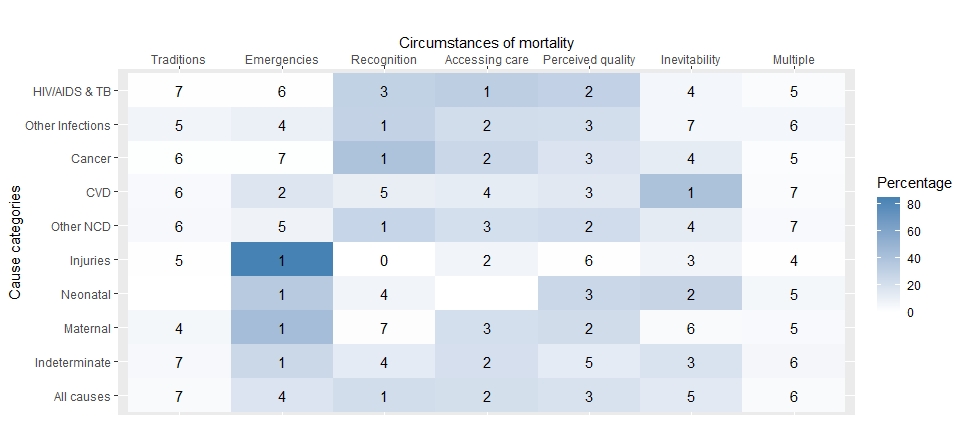


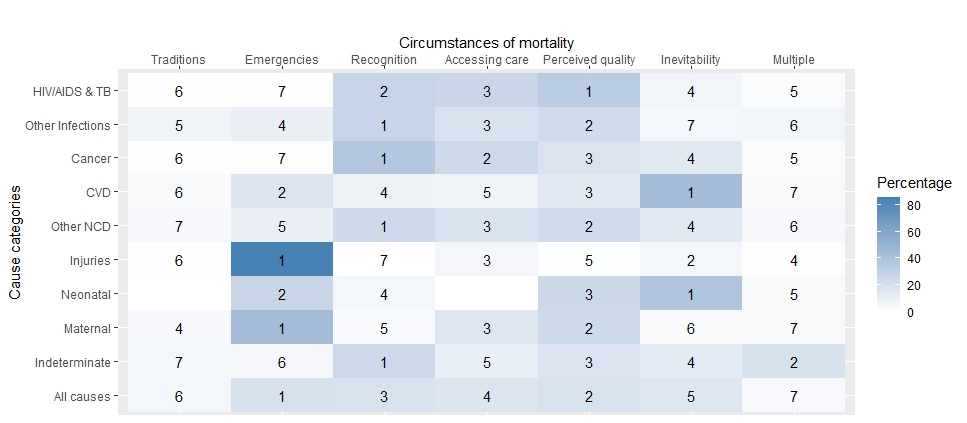
e: Assigned COMCATs ranked within each major cause of death category, for 3979 deaths in the Agincourt Health and Demographic Surveillance System (HDSS) 2012-16

f: Assigned COMCATs ranked within each major cause of death category for 5924 deaths in the Agincourt Health and Demographic Surveillance System (HDSS) 2012-19


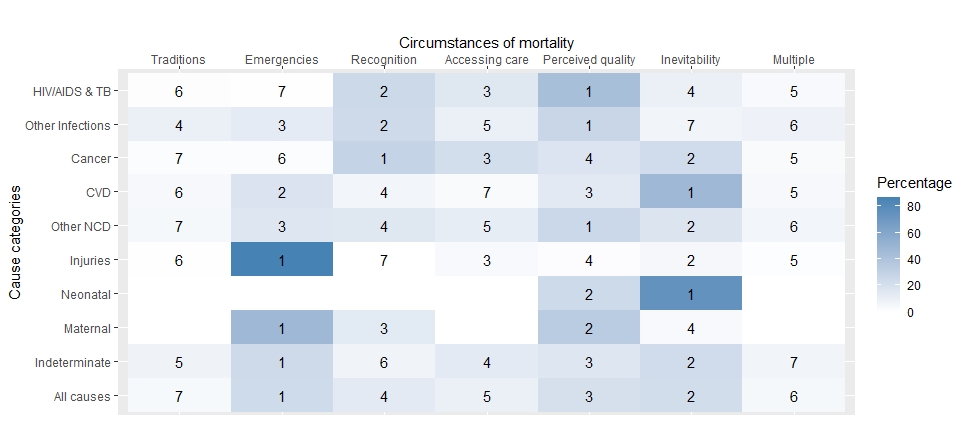


g: Assigned COMCATs ranked within each major cause of death category, for 1924 deaths in the Agincourt Health and Demographic Surveillance System (HDSS) 2017-19


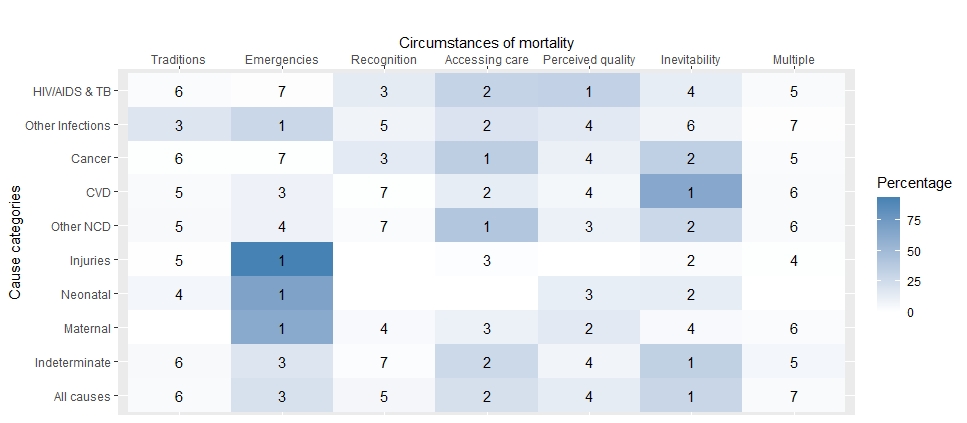


h: Assigned COMCATs ranked within each major cause of death category, for 2056 deaths in the AHRI Health and Demographic Surveillance System (HDSS) 2017-19


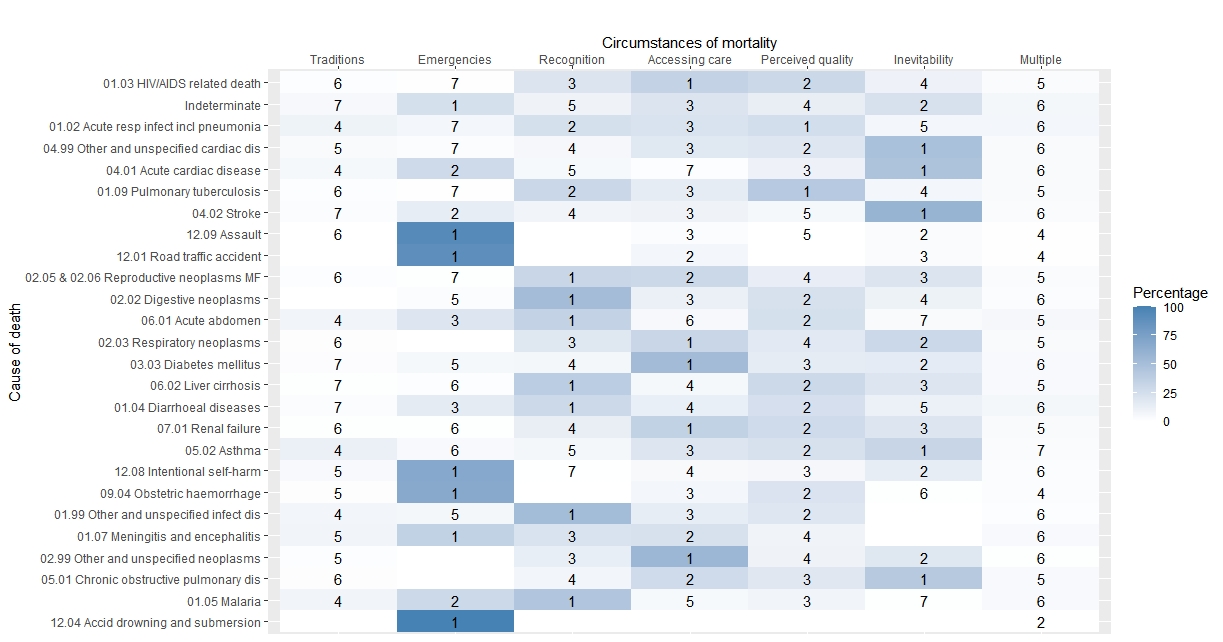


i: Assigned COMCATs ranked within causes of death accounting for 95% of the overall burden, for 7616 deaths in the Agincourt and AHRI Health and Demographic Surveillance Systems (HDSSs) 2012-19 and 2017-19
