# Supplementary material for: Refining circumstances of mortality categories (COMCAT): a verbal autopsy model connecting circumstances of deaths with outcomes for public health decision-making
Source: Glob Health Action. 2022 Apr 4;14(Suppl):2000091. doi: 10.1080/16549716.2021.2000091 (PMC8986216; doi:10.1080/16549716.2021.2000091)
Supplement: Supplemental Material [file ZGHA_A_2000091_SM0233.zip › z SM 2_Pathways R1.pptx]

## Slide 1
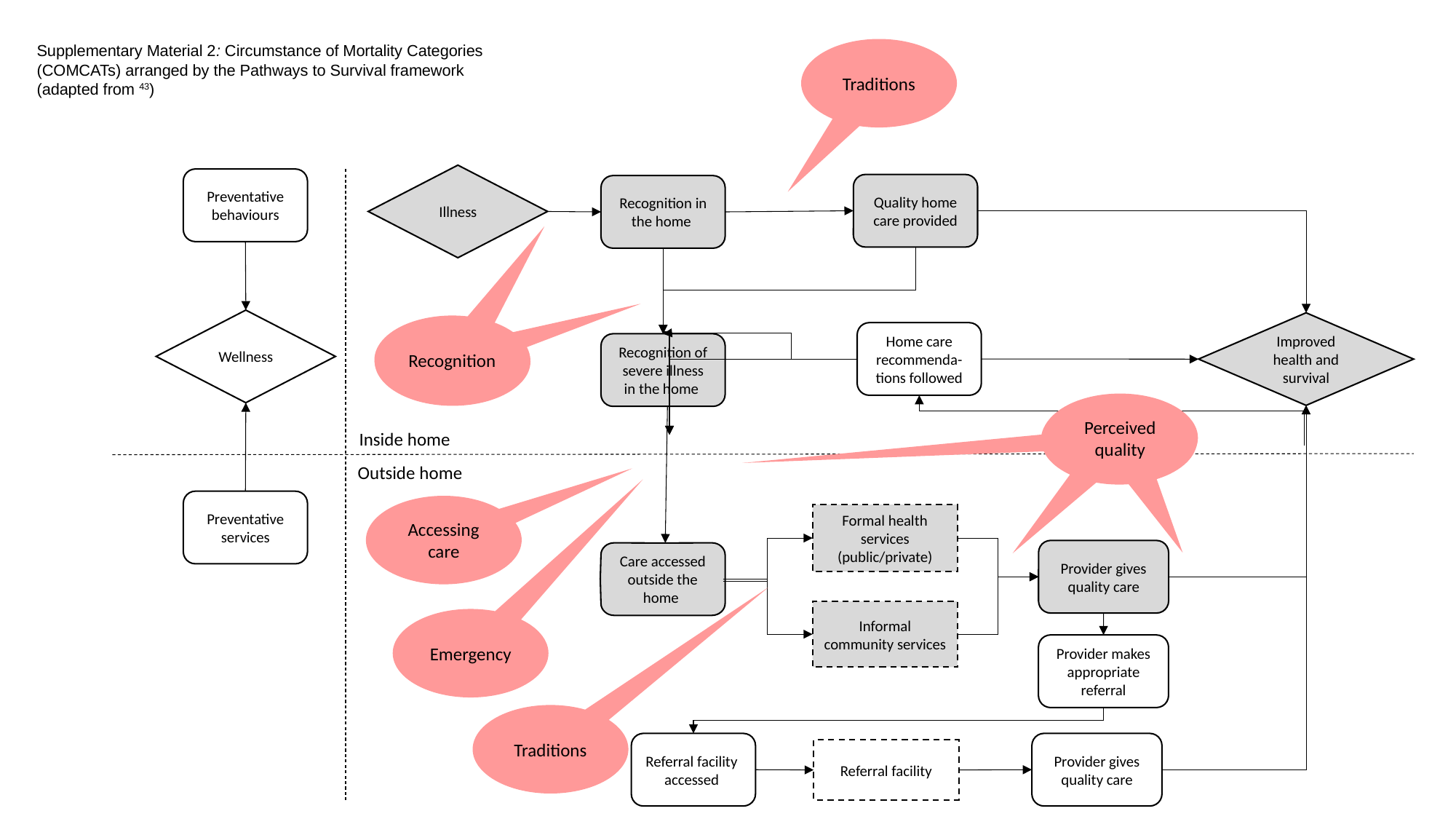

Supplementary Material 2: Circumstance of Mortality Categories (COMCATs) arranged by the Pathways to Survival framework (adapted from 43)
Traditions
Illness
Preventative behaviours
Quality home care provided
Recognition in the home
Wellness
Improved health and survival
Recognition
Home care recommenda-tions followed
Recognition of severe illness in the home
Perceived quality
Inside home
Outside home
Preventative services
Accessing care
Formal health services (public/private)
Provider gives quality care
Care accessed outside the home
Informal community services
Emergency
Provider makes appropriate referral
Traditions
Provider gives quality care
Referral facility accessed
Referral facility
