# Supplementary material for: Refining circumstances of mortality categories (COMCAT): a verbal autopsy model connecting circumstances of deaths with outcomes for public health decision-making
Source: Glob Health Action. 2022 Apr 4;14(Suppl):2000091. doi: 10.1080/16549716.2021.2000091 (PMC8986216; doi:10.1080/16549716.2021.2000091)
Supplement: Supplemental Material [file ZGHA_A_2000091_SM0233.zip › z SM 3_COM inputs assessment R1.docx]

Supplementary Material 3a: Assessment of circumstantial input indicators

| Circumstances of mortality inputs | Responses (1) | Use in model (2) | Importance (3) | Score (4) | Assessment | Proposed re-word |
| --- | --- | --- | --- | --- | --- | --- |
| *Existing input indicators* | | | | | | |
| Used traditional medicine | Low 1 | High 3 | High 3 | 7 | Retain | n/a |
| Costs prohibitive | Med 2 | Med 2 | High 3 | 7 | Retain | n/a |
| Hospital >2h | Low 1 | Med 2 | High 3 | 6 | Retain | n/a |
| Problems with treatment | Low 1 | Med 2 | High 3 | 6 | Retain | n/a |
| Problems with medication | Low 1 | Med 2 | High 3 | 6 | Retain | n/a |
| Travelled to hospital | Med 2 | Med 2 | High 3 | 7 | Reword | Was formal care sought in the final days? |
| Doubts about need for care | Low 1 | High 3 | High 3 | 7 | Reword | Was severity and need for care recognised? |
| Used motorised transport | Low 1 | Med 2 | High 3 | 6 | Reword | Were there problems arranging transport? |
| Used phone to call for help | Med 2 | Low 1 | Low 1 | 4 | Remove | n/a |
| Problems with admission | Low 1 | Med 2 | Low 1 | 4 | Remove | n/a |
| *Proposed replacement input indicators* | | | | | | |
| Referral made | - | - | - | - | Candidate replacement | Was the deceased referred to another place for care? |
| Referral reached | - | - | - | - | Candidate replacement | Did they reach the place they were referred to? |

(1) Responses ranked as: ‘low’ if substantive response identified in <10% of responses; ‘medium’ if substantive response identified in between >10% and <60%; and ‘high’ if substantive response identified in >60% of responses; (2) Use in model (predictive power) ranked ‘low’ if sum of probabilities linking the input to each COMCAT was 0.2 or less; medium if between >0.2 and 1.2; high if >1.3; (3) Importance ranked as likelihood of deriving important information across a range of LMIC settings through consensus between a group of experts in VA; (4) Summary score: sum of 1-3.


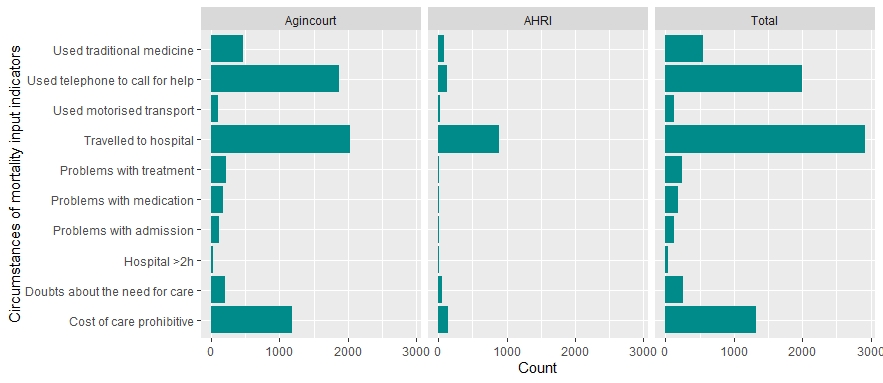


b: Use of circumstances of mortality input indicators: frequencies of substantive responses to 10 circumstantial input indicators for 7980 deaths in the Agincourt and AHRI Health and Demographic Surveillance System (HDSSs) 2012-19

c: Predictive power of circumstances of mortality input indicators: excerpt of matrix of conditional probabilities of symptoms (circumstantial input indicators) linked to circumstance of mortality categories (COMCATs)

| Circumstances of mortality inputs | Response | Traditions | Emergency | Recognition | Accessing care | Perceived quality | Inevitable |
| --- | --- | --- | --- | --- | --- | --- | --- |
| Used traditional medicine | Y | A+ | D | A | D | E | B |
| Used motorised transport | N | B | D | D | A | D | C |
| Used a telephone to call for help | N | C | C | B | B | D | C |
| Travelled to hospital | N | C | A | C | C | B | C |
| Problems with treatment | Y | C | D | C | D | A | B |
| Problems with medication | Y | D | C | D | B | A | B |
| Problems with admission | Y | C | E | C | D | A | C |
| Hospital >2 hrs | Y | C | A | C | C | B | C |
| Doubts about the need for care | Y | A | C | A+ | D | E | B |

| Label | Value | Interpretation |
| --- | --- | --- |
| I | 1.0 | Always |
| A+ | 0.8 | Almost always |
| A | 0.5 | Common |
| A- | 0.2 |  |
| B+ | 0.1 | Often |
| B | 0.05 |  |
| B- | 0.02 |  |
| C+ | 0.01 | Unusual |
| C | 0.005 |  |
| C- | 0.002 |  |
| D+ | 0.001 | Rare |
| D | 0.0005 |  |
| D- | 0.0001 |  |
| E | 0.0001 | Hardly ever |
| N | 0 | Never |
