# Supplementary material for: Refining circumstances of mortality categories (COMCAT): a verbal autopsy model connecting circumstances of deaths with outcomes for public health decision-making
Source: Glob Health Action. 2022 Apr 4;14(Suppl):2000091. doi: 10.1080/16549716.2021.2000091 (PMC8986216; doi:10.1080/16549716.2021.2000091)
Supplement: Supplemental Material [file ZGHA_A_2000091_SM0233.zip › z SM 4_COMCAT methods note R1.docx]

Supplementary Material 4:


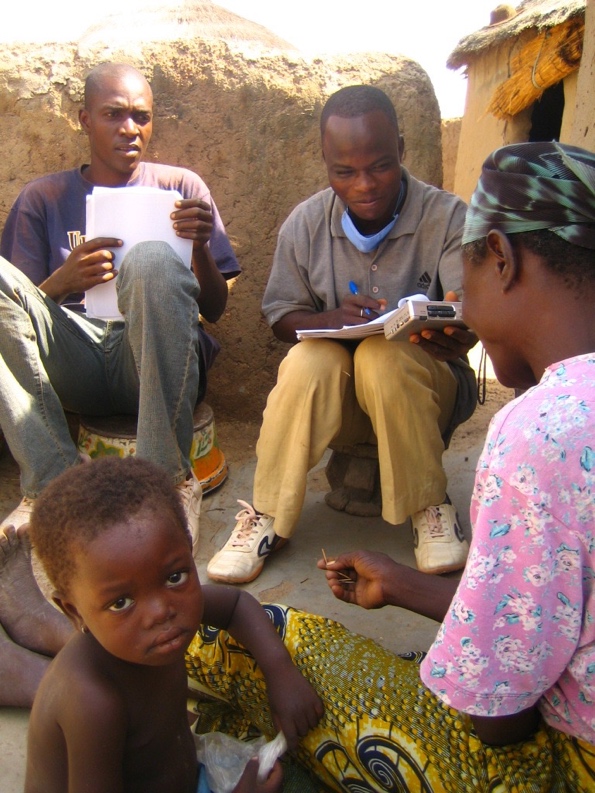


VA interview in Burkina Faso

(D’Ambruoso, 2008)

**How does the COMCAT model work**

**within Verbal Autopsy? An explanatory note**

***What is Verbal Autopsy?***

Verbal Autopsy (VA) is a survey method used to understand causes of death in populations. There are two main parts to VA: Firstly, structured interviews are conducted by trained fieldworkers with final caregivers of deceased persons on signs and symptoms and circumstances in the final days before death (see image).

Secondly, interview data are interpreted to arrive at probable cause(s) of death. Physicians traditionally interpreted VA interview data. New developments have enabled VA data to be interpreted by computers. Computer coding has been extensively tested and is 100% reliable and consistent, removing the need to use physician time. Interview data can also be collected on mobile devices and simultaneously fed into computer models producing likelihood scores for cause(s) of death.

***What is COMCAT?***

Circumstances of Mortality Categories (COMCAT) is a tool within VA computer coding that assigns circumstantial categories to deaths along with medical causes. COMCATs relate to critical limiting processes of care seeking and utilisation at and around time of death, and are intended to make distinctions between important circumstances of deaths.

For example, a woman whose cause of death is assigned as obstetric haemorrhage, might have died at home because she had no means to travel to a facility, while another woman with the same cause of death might have been inadequately managed despite getting to a health facility. Differentiating between such scenarios is important for planning and evaluating health services and reducing avoidable mortality.

While the social determinants of health have been widely accepted as the fundamental root causes of avoidable mortality. Systematic, consistent and scalable attribution of circumstantial categorisations of deaths has not been developed or used. The COMCAT system enables analysis of circumstances of death as a routine analytic component in addition to medical cause of death for service organisation and delivery.

| COMCATs | Description of circumstantial category |
| --- | --- |
| Traditions | Traditional practices or beliefs influenced health seeking behaviour and the pathway to death |
| Emergencies | Sudden, urgent or unexpected conditions leading to death |
| Recognition | Lack of recognition or awareness of serious disease (e.g., symptoms or severity) negatively influenced health seeking behaviour |
| Accessing care | Inability to mobilise and use resources (e.g., material, transport, financial) hindered access to health care |
| Perceived quality | Problems in getting health care despite accessing health facilities (e.g., related to admissions, treatments and medications) |
| Referral | Problems receiving a referral when required. Problems reaching referral facility after referral made |
| Inevitability | Death occurred in circumstances that could not reasonably have been averted (e.g., very elderly or recognised terminal conditions) |
| Multiple | A combination of the above categories affected the pathway to death; no single factor predominated |

***What are COMCATs used for?***

COMCAT is not intended to replace critical incident review or other facility based audits involving in-depth investigations of cases and outcomes. The system is instead developed for large scale population assessment and is aimed at health service managers and planners to monitor population health and support assessment of services and evaluations encompassing medical, social and health systems aspects.

***How are COMCAT data visualised?***

*
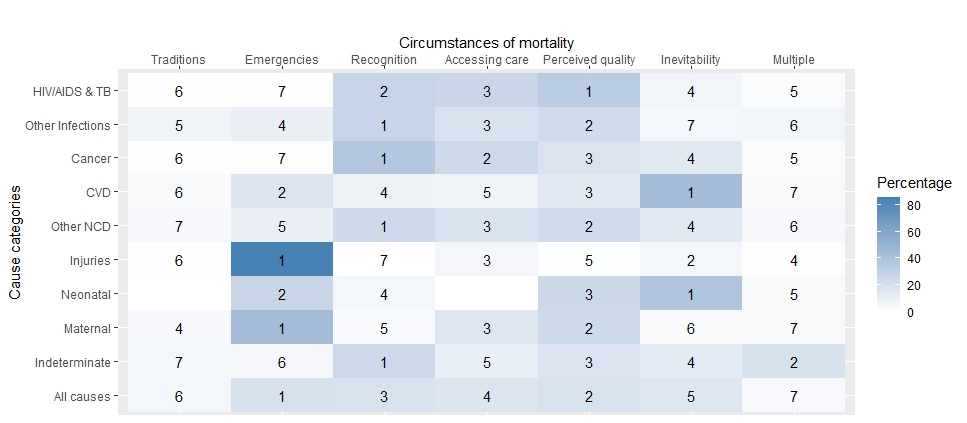
*Data can be visualised in different ways to understand caregiver perspectives on the needs, perceptions and behaviours of individuals, as well as the responsiveness of the health system towards these. The image below shows COMCATs ranked within each cause of death, for deaths in a representative surveillance area in Mpumalanga 2012-19. This indicates, for example, that deaths attributed to injuries are predominately associated with the COMCAT ‘emergencies’ and HIV/AIDS and TB deaths with ‘perceived quality’.

The second image below shows the number of deaths in the same surveillance area in Mpumalanga 2012-19 attributed to HIV/AIDS, STIs and TB (left hand side) and COMCATs (right hand side) both arranged by year and age groups. This visualisation shows trends over time in outcomes and circumstantial drivers of outcomes. While HIV/AIDS, STIs and TB deaths, and the circumstances of HIV/AIDS, STIs and TB deaths, are decreasing over time, problems with ‘perceived quality’ continues to characterise HIV/AIDS, STIs and TB deaths.


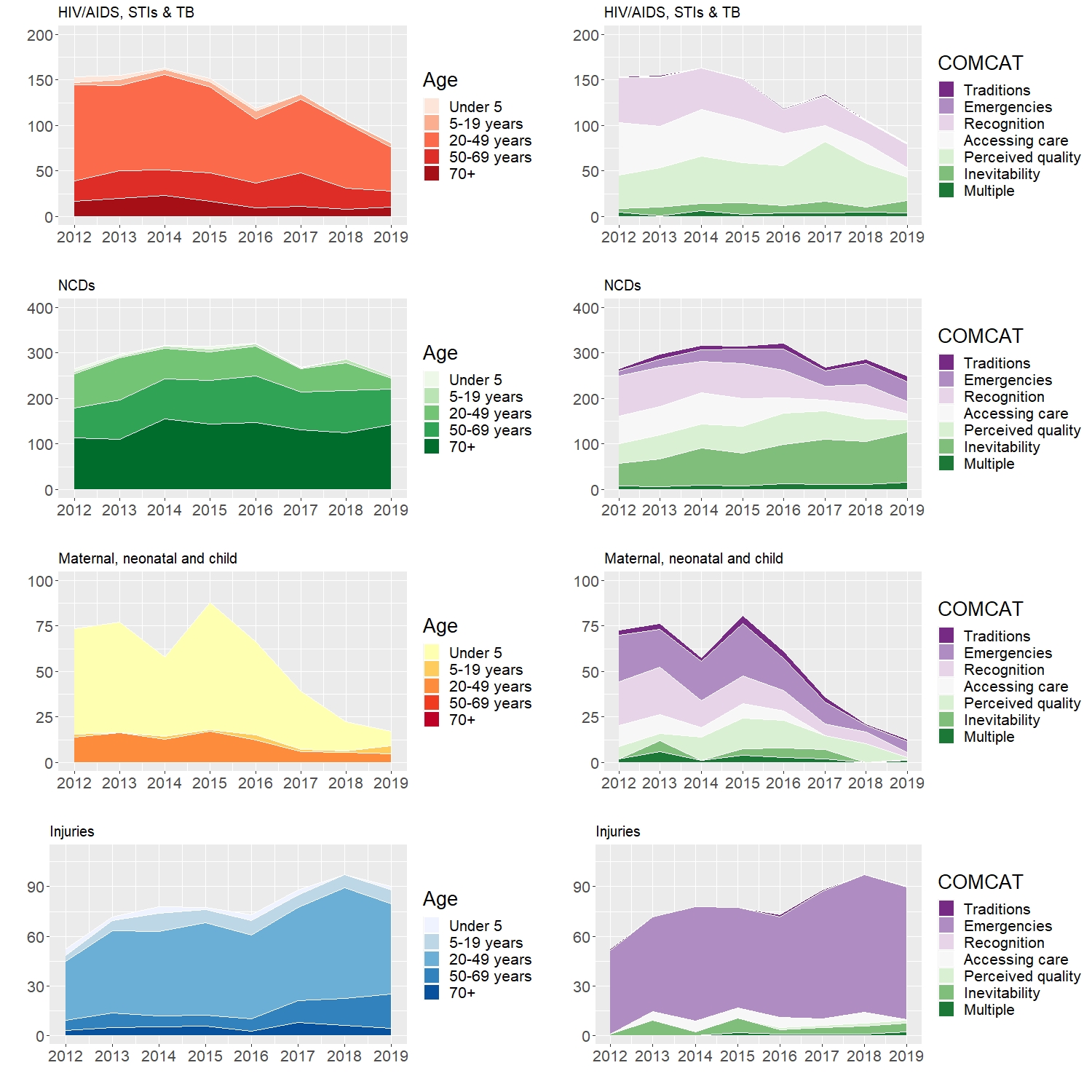


***How are data used?***

COMCATs can help establish an impetus for action and investment and can be operationalised into targets for intervention that correspond with programme areas were seen as highly relevant. For example, where ‘emergencies’ is a significant factor contributing to maternal and neonatal deaths, improving emergency medical services including transport would be indicated to reduce deaths in this category.

Both VA and COMCAT are tools capable of making contributions in an overall toolbox of data and the greatest benefit is when different tools are used. Specifically, the District Health Information System (DHIS) provides a facility-based picture, while VA and COMCAT add a social and health systems dimension and provide an overall account inclusive of the community deaths. Together, these tools are complementary.

***Further information***

The data were developed by researchers from the Verbal Autopsy with Participatory Action Research (VAPAR) programme at the University of the Witwatersrand and the South African Population Research Infrastructure Network (SAPRIN). Please get in touch if you would like more information [contact details].
