# Supplementary material for: Refining circumstances of mortality categories (COMCAT): a verbal autopsy model connecting circumstances of deaths with outcomes for public health decision-making
Source: Glob Health Action. 2022 Apr 4;14(Suppl):2000091. doi: 10.1080/16549716.2021.2000091 (PMC8986216; doi:10.1080/16549716.2021.2000091)
Supplement: Supplemental Material [file ZGHA_A_2000091_SM0233.zip › z SM 5_COMCAT visualisations R1.pptx]

## Slide 1
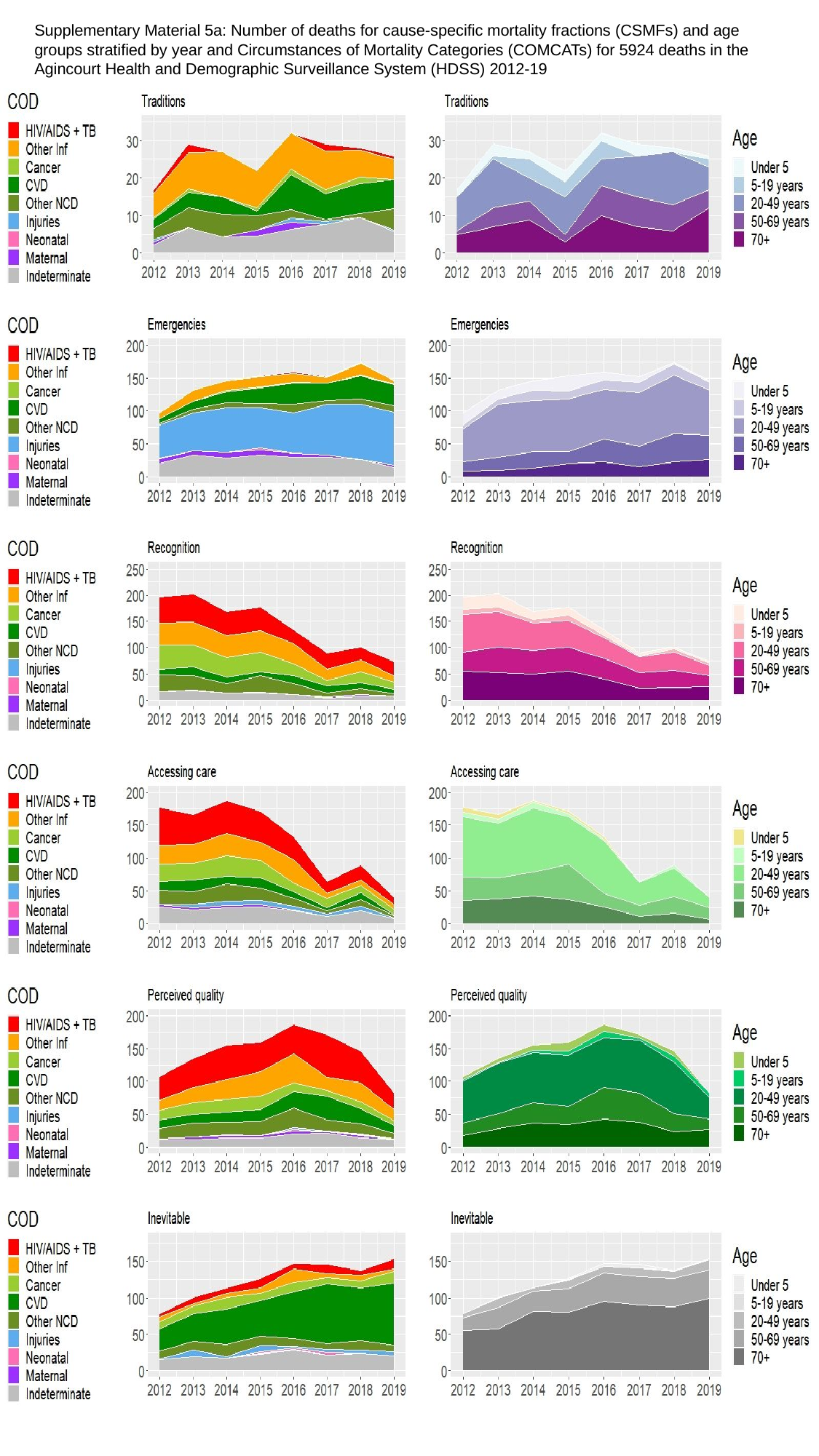

Supplementary Material 5a: Number of deaths for cause-specific mortality fractions (CSMFs) and age groups stratified by year and Circumstances of Mortality Categories (COMCATs) for 5924 deaths in the Agincourt Health and Demographic Surveillance System (HDSS) 2012-19

## Slide 2
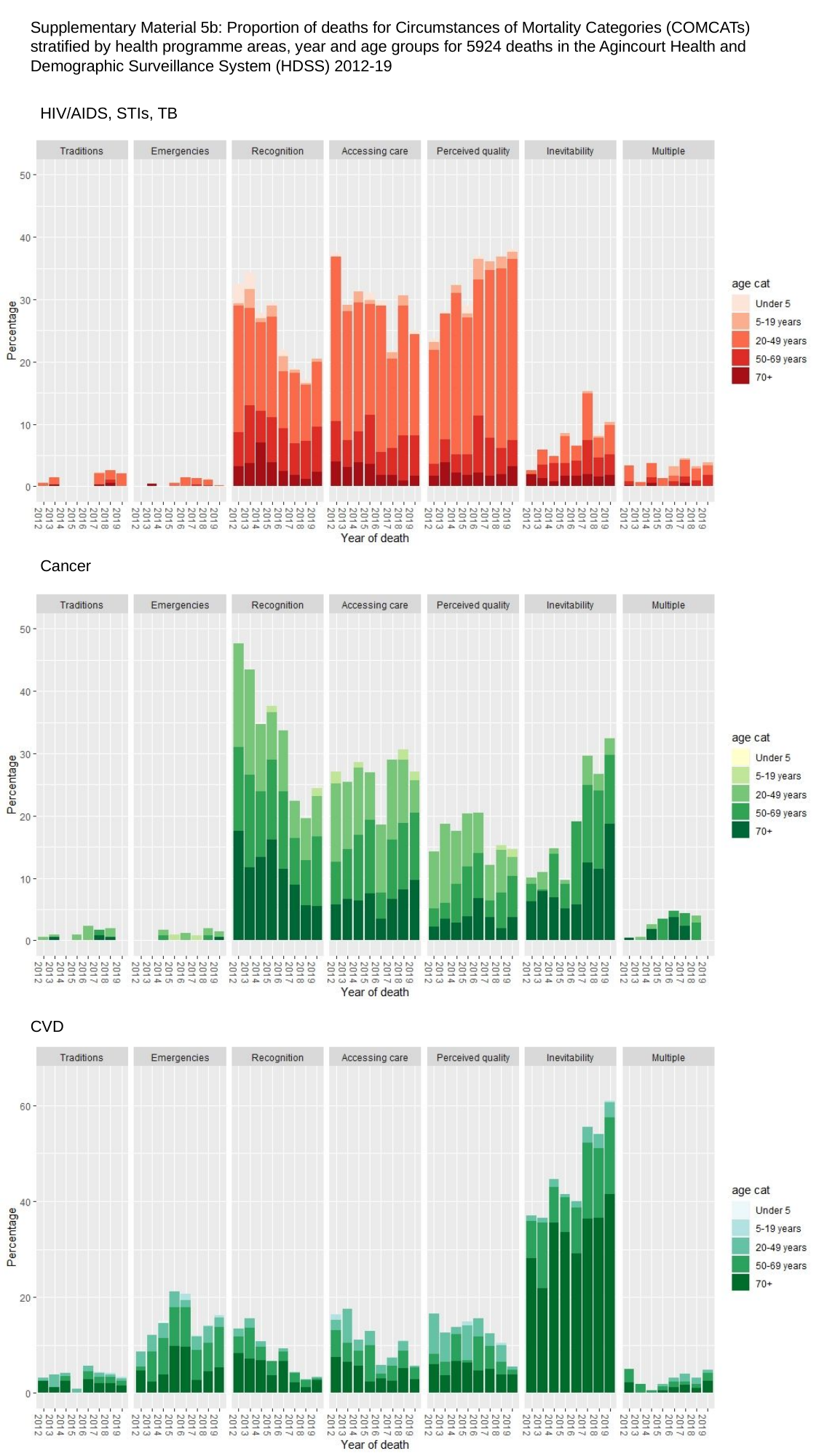

Supplementary Material 5b: Proportion of deaths for Circumstances of Mortality Categories (COMCATs) stratified by health programme areas, year and age groups for 5924 deaths in the Agincourt Health and Demographic Surveillance System (HDSS) 2012-19
HIV/AIDS, STIs, TB
Cancer
CVD

## Slide 3
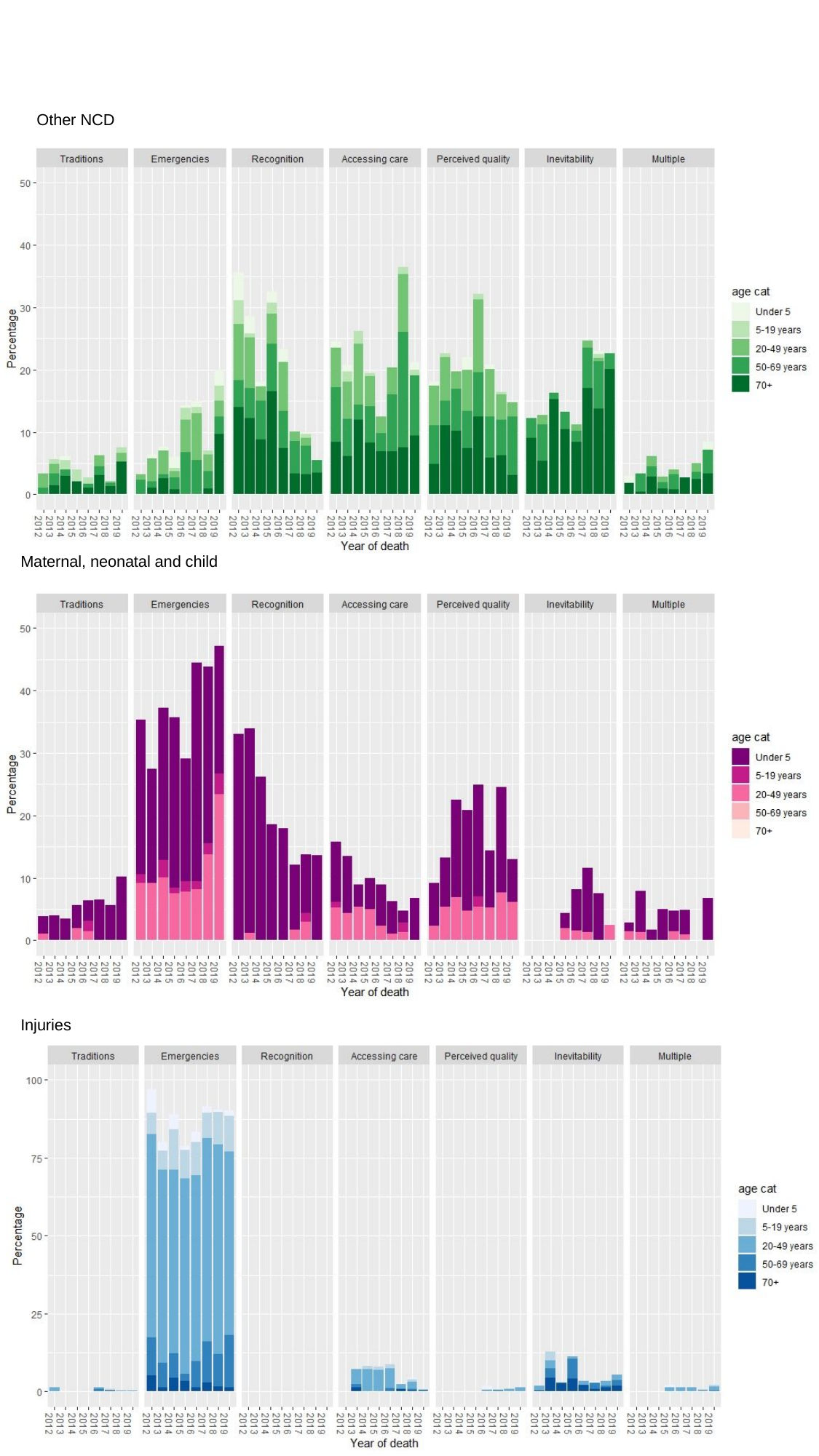

Other NCD
Maternal, neonatal and child
Injuries
